# The Alternative Sigma Factor SigL Influences *Clostridioides difficile* Toxin Production, Sporulation, and Cell Surface Properties

**DOI:** 10.3389/fmicb.2022.871152

**Published:** 2022-05-11

**Authors:** Andrew E. Clark, Chelsea C. Adamson, Katelyn E. Carothers, Bryan Angelo P. Roxas, V. K. Viswanathan, Gayatri Vedantam

**Affiliations:** ^1^School of Animal and Comparative Biomedical Sciences, Tucson, AZ, United States; ^2^Department of Immunobiology, University of Arizona, Tucson, AZ, United States; ^3^BIO5 Institute for Collaborative Research, University of Arizona, Tucson, AZ, United States; ^4^Southern Arizona VA Healthcare System, Tucson, AZ, United States

**Keywords:** *Clostridium difficile*, SigL, sigma 54, RpoN, sporulation, *Clostridioides difficile*

## Abstract

The alternative sigma factor SigL (Sigma-54) facilitates bacterial adaptation to the extracellular environment by modulating the expression of defined gene subsets. A homolog of the gene encoding SigL is conserved in the diarrheagenic pathogen *Clostridioides difficile*. To explore the contribution of SigL to *C. difficile* biology, we generated *sigL*-disruption mutants (*sigL::erm*) in strains belonging to two phylogenetically distinct lineages—the human-relevant Ribotype 027 (strain BI-1) and the veterinary-relevant Ribotype 078 (strain CDC1). Comparative proteomics analyses of mutants and isogenic parental strains revealed lineage-specific SigL regulons. Concomitantly, loss of SigL resulted in pleiotropic and distinct phenotypic alterations in the two strains. Sporulation kinetics, biofilm formation, and cell surface-associated phenotypes were altered in CDC1 *sigL::erm* relative to the isogenic parent strain but remained unchanged in BI-1 *sigL::erm*. In contrast, secreted toxin levels were significantly elevated only in the BI-1 *sigL::erm* mutant relative to its isogenic parent. We also engineered SigL overexpressing strains and observed enhanced biofilm formation in the CDC1 background, and reduced spore titers as well as dampened sporulation kinetics in both strains. Thus, we contend that SigL is a key, pleiotropic regulator that dynamically influences *C. difficile*'s virulence factor landscape, and thereby, its interactions with host tissues and co-resident microbes.

## Introduction

*Clostridioides difficile*, an anaerobic, spore-forming, Gram-positive bacillus, is a significant cause of healthcare-associated infections (Stewart et al., 2020). *C. difficile* infection manifests as a diverse spectrum of diarrheal diseases with possible progression toward more serious sequelae, including pseudomembranous colitis or fatal toxic megacolon (George et al., 1978; Dudukgian et al., 2009). Ingested spores germinate into vegetative cells upon exposure to specific bile salt and amino acid triggers in the mammalian host. Disruption or suppression of the endogenous microbiota by antibiotics, immunosuppressive drugs, or host factors, facilitate vegetative cell colonization of the intestine. Vegetative cells from toxigenic *C. difficile* strains produce two major enterotoxins (TcdA and TcdB) that glucosylate host Rho GTPases, leading to actin cytoskeleton reorganization, solute transport disruption, and consequent symptomatic disease (Vedantam et al., 2012). *C. difficile* toxin synthesis increases as bacteria enter the stationary phase of growth; thus pathogen metabolism and virulence are linked (Voth and Ballard, 2005; Dineen et al., 2010; Bouillaut et al., 2015).

Sigma factors are dissociable components of the RNA polymerase holoenzyme complex that recognize and bind defined promoter sequences, thereby dictating cellular transcriptional programs (Merrick, 1993; Feklístov et al., 2014). Canonical sigma-70 family members engage promoters at −35 and −10 regions relative to the transcriptional start site, induce promoter open-complex formation, and initiate transcription (Zhang and Buck, 2015). In contrast, the structurally unrelated sigma-54 family members recognize unique −24 and −12 promoter regions; however, the formation of a repressive fork junction structure near −12 prevents the recruited holoenzyme from forming an open complex unassisted (Rappas et al., 2007; Zhang and Buck, 2015). Conversion to an open complex requires energy transfer from unique bacterial enhancer-binding proteins (EBPs, or sigma-54 activators) that bind conserved enhancer sequences upstream of the promoter. The formation of a hairpin-like structure, assisted by DNA-bending proteins, such as integration host factor (IHF), allows enhancer-bound EBPs to interact with the promoter-bound sigma-54 closed complex (Zhang and Buck, 2015). Activator-mediated ATP hydrolysis results in loss of the fork junction structure and isomerization to an open complex, leading to transcription initiation.

Nie et al. used comparative genomics of 57 species from the *Clostridiales* to reconstruct sigma-54 regulons and identify EBPs and their regulatory modules (Nie et al., 2019). Of the 24 predicted EBPs detected in *C. difficile*, the roles of two, CdsR and PrdR, involved in cysteine and proline catabolism, respectively, were experimentally validated (Bouillaut et al., 2013; Gu et al., 2017). To comprehensively characterize the SigL regulon, Soutourina et al. used *in silico* analysis, transcriptome analyses, and transcription start site (TSS) mapping of the laboratory-adapted *C. difficile* strain 630Δ*erm* (Soutourina et al., 2020). These studies revealed that the SigL regulon includes genes involved in the oxidation and reduction of amino acids to the corresponding organic acids (Stickland metabolism). Stickland reactions generate ATP and NAD^+^ and regulate *C. difficile* toxin production and virulence (Stickland, 1934; Neumann-Schaal et al., 2019).

To expand these observations to clinically-contemporary isolates, and further characterize the role of SigL in *C. difficile* biology, we generated isogenic insertion mutants (*sigL::erm*) in two genetically-divergent strains of human and veterinary origin: BI-1 (flagellated) and CDC1 (non-flagellated), belonging to the lineages (Ribotypes) RT027 and RT078, respectively. The epidemic-associated RT027 strains are often responsible for the most severe human *C. difficile* infections (CDI). RT078 strains, in contrast, while sharing several attributes with RT027 strains, are frequently associated with livestock infections (Elliott et al., 2017). We performed phenotypic assays and comparative proteomics studies on parental and *sigL::erm* derivatives. SigL modulated the *C. difficile* proteome in a temporal manner, and loss of *sigL* had profound but contrasting and pleiotropic effects on the two strains. Our studies support a key role for SigL in *C. difficile* gene regulation, including strain-specific roles in the modulation of virulence factor expression, central metabolic functions, and sporulation.

## Methods

### Bacterial Strains and Growth Conditions

All *C. difficile* strains utilized in this study (Supplementary Table S4) were grown at 37°C using a Type B Coy Laboratory Anaerobic Chamber under anaerobic conditions in an 85% N_2_, 10% H_2_, 5% CO_2_ gas mixture. Bacteria were recovered from the freezer storage in glycerol and routinely maintained on BHIS agar plates (Brain-Heart Infusion base media supplemented with 5 g/L Yeast Extract and 0.1% L-Cysteine). All assays requiring liquid culture were performed using Bacto™ BHI unless otherwise indicated.

Antibiotics were used as necessary for *C. difficile*: (15 μg/mL thiamphenicol, 20 μg/mL lincomycin, 0.8 μg/mL cefoxitin, 25 μg/mL cycloserine) added to either BHI or BHIS. *E. coli* Top10 (Invitrogen) used for cloning and plasmid construction, and CA434 used for conjugation were routinely grown at 37°C on LB media using antibiotics as necessary: (20 μg/mL chloramphenicol, 100 μg/mL spectinomycin, 150 μg/ml ampicillin). The *C. difficile* clinical isolates used in this study were from the Hines VA Hospital culture collection of Dr. Dale Gerding or from the collection of Dr. Glenn Songer.

### *In silico sigL* Sequence, Promoter, and Functional Domain Analysis

*C. difficile* genome sequences were maintained and annotated using Rapid Annotation using Subsystem Technology (RAST) bioinformatics web tools (Overbeek et al., 2014). *sigL* promoter prediction reported here was undertaken using Microbes Online web tools (Dehal et al., 2010). Functional domains of *C. difficile* SigL reported here were analyzed using the Similar Modular Architecture Research Tool (SMART) (Letunic et al., 2015).

### Quantitative Real-Time PCR

*C. difficile* BI-1 and CDC1 strains were grown to mid-exponential (OD600 ~ 0.6), late-exponential (OD600 ~ 0.8), and stationary phase (OD600 ~ 1.0). At each phase of growth, cultures were diluted 1:2 in RNA*later* RNA stabilization reagent (Sigma Aldrich) and incubated anaerobically for 1 h, before pelleting and freezing bacteria at −80°C. RNA was extracted from cell pellets using the RNeasy Plus Mini Kit (Qiagen) according to manufacturer instructions, with modifications. Briefly, beat-beading tubes were filled 1/3 with beads and soaked overnight in 500 μl buffer RLT (Qiagen). Tubes were spun at full speed and excess buffer was removed. Bacterial pellets were lysed by bead beating for 2 min in 600 μl buffer RLT and 2-Mercaptoethanol (10 μl/mL) per sample. Tubes were centrifuged at max speed for 3 min, and the supernatant was removed for RNA extraction according to manufacturer instructions. RNA samples were treated with DNase I to remove genomic DNA contamination, and RNA yields were quantified on a Nanodrop instrument. cDNA synthesis from the RNA samples was performed using the SuperScript III First-Strand Synthesis System (Invitrogen) according to manufacturer instructions and yields were quantified using Nanodrop. Standard PCR was performed to confirm the expression of *sigL* and *16S* (housekeeping gene), with Taq 2× Master Mix, using AC106 *sigL*-F/AC106 *sigL*-R and *16S*RT_F/*16S*RT_R primer pairs respectively (Supplementary Table S5). Quantitative real-time PCR was performed using iTaq Universal SYBR Green Supermix (BioRad) on the StepOnePlus instrument (Applied Biosystems) according to manufacturer instructions. Expression fold changes were determined using ΔΔCT calculations (Livak and Schmittgen, 2001).

### Generation and Confirmation of *sigL* Mutants

Isogenic *sigL* mutants were created in *C. difficile* BI-1 and CDC1 using the ClosTron system (Kuehne and Minton, 2012). Briefly, retargeted ClosTron introns were designed using the Perutka algorithm at Clostron.com and synthesized by DNA2.0 (DNA2.0, Melo Park, CA). The resulting constructs were electrotransformed into *E. coli* CA434 donor strain which was recovered on LB supplemented with spectinomycin and chloramphenicol. Isolated colonies were inoculated into BHIS broth supplemented with chloramphenicol and grown overnight at 37°C with shaking. One milliliter of culture was pelleted at 1,500 × g for 1 min, washed in BHIS, centrifuged again for 1 min at 1,500 × g, and transferred to an anaerobic chamber. The pellet was subsequently resuspended with 200 μl of *C. difficile* overnight recipient culture and incubated overnight on BHIS agar. Growth was recovered in one milliliter of PBS, and 200 μl aliquots were inoculated to BHIS Tetrazolium Chloride (TCC) and incubated overnight at 37°C. Thiamphenicol-resistant colonies were isolated on BHIS supplemented with lincomycin to isolate potential integrant clones. Integration of the intron was confirmed by PCR using primers that detect both the integrated form of the intron, as well as primers AC55 *sigL*-F and AC56 *sigL*-R which amplify the intron/*sigL* junction (Supplementary Table S5, Supplementary Figure S2). Loss of SigL expression was verified by RT-PCR using primers AC106 *sigL*-F and AC107 *sigL*-R (Supplementary Figure S2), and *sigL* disruption mutants were cured of the Clostron plasmid by serial passage on BHIS without selection.

### Generation of Complemented and *sigL*-Overexpressing Strains

The pMTL8000-series of shuttle vectors was used for the construction of *sigL* complementation and overexpression plasmids. Primers AC55 *sigL*-F and AC56 *sigL*-R were designed to amplify the coding sequence ~300 base pairs upstream of the *sigL* gene. The resulting PCR products were cleaned and digested with restriction enzymes allowing them to be cloned into either pMTL82151 and expressed under the control of their endogenous promoter (complementation) or pMTL82153 and expressed under the control of a constitutive promoter (Pfdx, overexpression). pMTL82151 and pMTL82153 contain the same backbone, including the Gram(+)Rep (pBP1), marker (*catP*), and Gram(–)Rep (ColE1+tra). The resulting plasmids were transformed into CA434 and conjugated into *C. difficile sigL* mutants. Successfully complemented strains were selected on BHIS containing thiamphenicol.

### *In silico* Analysis of SigL-Dependent Promoters

Twelve putative promoter sequences for genes or operons with previously established downregulation in *sigL* mutants (Soutourina et al., 2020) were used to generate a positional weight matrix (PWM). Genomes of 630 (NCBI Assembly GCA_000009205.2), BI-1 (GCA_000211235.1), and an RT078 strain, QCD-23m63 (GCA_000155065.1), were searched for promoter sites based on the *C. difficile sigL* PWM using FIMO (Find Individual Motif Occurrences) (Grant et al., 2011). Identified *sigL* sites in 630, BI-1, and RT078 with FIMO q-values <0.05 located <200 bp upstream of the gene and/or operon were compiled, tabulated, and cross-referenced to previous *C. difficile* 630 *sigL* promoters, enhancer-binding protein (EBP) upstream activator sequences (UAS), and *sigL::erm* microarray results (Nie et al., 2019; Soutourina et al., 2020). The sequence and position of EBP UAS identified by Nie et. al. in BI-1 and RT078 genomes were determined using BLASTn.

### Protein Processing and iTRAQ Labeling

*C. difficile* was grown to an OD_600_ of either 0.6 (mid-exponential), 0.8 (late-exponential) or 1 (stationary) in BHI. Cell pellets were resuspended in 400 μl of 0.5 M triethylammonium bicarbonate (TEAB) buffer and sonicated (five pulses of 15 s duration each) at an amplitude setting of 4 for lysis. Lysates were centrifuged at 20,000 × g for 30 min at 4°C to clear debris. Total recovered proteins were quantitated using a BCA Protein Assay (Pierce, Rockford, IL).

A total of 100 μg of protein in 20 μl of 0.5 M TEAB from each sample was denatured (1 μl of 2% SDS), reduced (1 μl of 100 mM tris-(2-carboxyethyl) phosphine), and alkylated (1 μl of 84 mM iodoacetamide). Trypsin was added at a ratio of 1:10 and the digestion reaction was incubated for 18 h at 48°C. iTRAQ reagent labeling was performed according to the 8-plex iTRAQ kit instructions (AB SCIEX, Framingham, MA).

### 2D-LC Fractionation

Strong cationic exchange (SCX) fractionation was performed on a passivated Waters 600E HPLC system, using a 4.6 × 250 mm polysulfoethyl aspartamide column (PolyLC, Maryland, USA) at a flow rate of 1 ml/min. Fifteen SCX fractions were collected and dried down completely, then resuspended in 9 μl of 2% (v/v) acetonitrile (ACN) and 0.1% (v/v) trifluoroacetic acid (TFA).

For the second-dimension fractionation by reverse phase C18 nanoflow-LC, each SCX fraction was autoinjected onto a Chromolith CapRod column (150 × 0.1 mm, Merck) using a 5 μl injector loop on a Tempo LC MALDI Spotting system (ABI-MDS/Sciex). Separation was over a 50 min solvent gradient from 2% ACN and.1% TFA (v/v) to 80% ACN and 0.1% TFA (v/v) with a flow rate of 2.5 μl/min. An equal flow of MALDI matrix solution was added post-column (7 mg/ml recrystallized CHCA (α-cyano-hydroxycinnamic acid), 2 mg/ml ammonium phosphate, 0.1% TFA, 80% ACN). The combined eluent was automatically spotted onto a stainless steel MALDI target plate every 6 s (0.6 μl per spot), for a total of 370 spots per original SCX fraction.

### Mass Spectrometry Analysis and Protein Quantitation

MALDI target plates were analyzed in a data-dependent manner on an ABSciexTripleTOF 5,600+ mass spectrometer (AB SCIEX, Framingham, MA). MS spectra were acquired from each sample spot using 500 laser shots per spot, laser intensity of 3,200. The highest peak of each observed m/z value was selected for subsequent MS/MS analysis.

Up to 2,500 laser shots at laser power 4,200 were accumulated for each MS/MS spectrum. Protein identification and quantitation were performed using the Paragon algorithm implemented in Protein Pilot 3.0 software by searching the acquired MS and MS/MS spectra from all 15 plates against the *C. difficile* strain BI-1 protein database, or the 078 representative strain QCD-23m63 database, plus common contaminants. The Protein Pilot Unused score cutoff of >0.82 (1% global false discovery rate) was calculated from the slope of the accumulated Decoy database hits by the Proteomics System Performance Evaluation Pipeline (PSPEP) program [72]. Proteins with at least one peptide >95% confidence (score based on the number of matches between the data and the theoretical fragment ions) and a Protein Pilot Unused score of >0.82 were considered as valid identifications (IDs).

### Identification of Differentially Abundant Proteins

A pooled sample consisting of equal amounts of each protein sample was used as a technical replicate. iTRAQ tags 113 and 121 were used to tag equal amounts of the same pooled sample. A receiver operating characteristic (ROC) curve was plotted using the technical replicate ratios, 119:121 and 121:119, as true negatives and all studied ratios as true positives. A 2-fold change cut-off was set to define proteins altered in abundance between wt and respective *sigL::erm* mutants.

### PSII Extraction and Detection

PSII extraction and quantitation were performed by previously published methods (Chu et al., 2016) with minor modifications. *C. difficile* vegetative cells were grown to an OD_600_ = 1.2 in BHI at 37°C. Cells were pelleted by centrifugation for 10 min at 4,000 rpm, and the supernatant was discarded. Pellets were resuspended in 0.1 M EDTA-triethanolamine buffer pH 7.0 for 20 min, and subsequently centrifuged for 10 min at 4,000 rpm. The supernatant was collected, serially diluted, and dot-blotted onto activated PVDF membranes, and probed with PSII-specific antiserum. Antibodies were detected using anti-Rabbit HRP secondary and FemtoWest Maximum sensitivity substrate (Pierce).

### Autolysis Assays

Autolysis of wild-type and *sigL* mutant strains was measured using the method described previously (El Meouche et al., 2013). Fifty milliliters *C. difficile* cultures were grown in BHI to an OD_600_ = 1.2 and were centrifuged for 10 min at 3,200 × g to pellet. The harvested cells were washed twice in PBS and resuspended in 50 ml of 50 mM phosphate buffer containing 0.01% of Triton X-100. Absorbance values at OD_600_ of resuspended cells were recorded, and cultures were placed back into an anaerobic environment at incubated at 37°C. Time points were harvested every 15 min, and autolysis was monitored by measuring absorbance values as a percentage of the initial OD_600_. Each assay was performed in biological triplicate and photographed at the termination of the experiment.

### Autoaggregation Analysis

Autoaggregation was measured by the method of Faulds-Pain et al. (2014) with modifications. Overnight cultures of *C. difficile* wild type and *sigL* mutants were diluted at 1:50 in BHI, and 5 ml of culture was separated into pre-reduced glass culture tubes and incubated upright at 37°C. Each hour, six culture tubes were removed from incubation and the OD_600_ was determined. The OD_600_ was directly read from the top 1 mL of culture in three “un-mixed” fractions per time point, and the remaining three tubes were vortexed and the 0D_600_ was measured constituting “mixed” fractions. Aggregated cells settle from suspension and are not recorded in the OD_600_ from “unmixed” fractions but are detected in the “mixed” fractions. The percent agglutination of aggregative *C. difficile* vegetative cells was calculated as the difference between the OD_600_ of the whole (mixed) solution and the OD_600_ of the top 1 ml (unmixed) as a percentage of the whole (mixed) solution OD_600_.

### Biofilm Assays

The influence of SigL expression on *C. difficile* biofilm formation was evaluated using a biofilm assay as previously reported (Pantaléon et al., 2015) with modifications. Overnight *C. difficile* cultures were diluted 1:50 in BHI broth and distributed into 12-well plates, 2 ml per well. Plates were wrapped to prevent evaporation and incubated anaerobically at 37°C for 72 h. Following incubation, the culture supernatant was removed and the biofilms were washed twice with PBS and then incubated at 37°C with 0.2% crystal violet to fix and stain. The crystal violet was subsequently removed, and the biofilms were washed an additional two times with PBS and photographed. To measure biofilm formation, crystal violet retained by the biomass was released with acetone/ethanol and quantitated by taking absorbance measurements at 570 nm in a technical quadruplicate for three individual wells.

### *C. difficile* Adhesion Assays to Caco2-BBE Human Intestinal Epithelial Cells

*C. difficile* attachment to human colonic epithelial cells was performed using the modified methods of Merrigan et al. (2013). C2BBe1 cells were obtained from the American Type Culture Collection (ATCC) and were grown to confluent monolayers in 5% CO_2_ in DMEM +10% fetal bovine serum, 20 μM HEPES, and 100 IU/mL Penicillin and Streptomycin in 6 well tissue culture plates. Twenty-four hours prior to the attachment assay, cells were washed and serum-free DMEM was applied. All additional assay solutions used were pre-reduced in the anaerobic chamber for at least 14 h. *C. difficile* strains to be analyzed were subcultured from overnight growth in BHI 1:50 into BHI and grown to an OD_600_ = 0.4 under anaerobic conditions. C2BBe1 cells were enumerated and multiplicity of infection (MOI) was calculated for each individual strain tested with a target of MOI = 50. *C. difficile* was harvested at OD_600_ = 0.4 and centrifuged for 5 min at 3,200 × g to remove BHI. *C. difficile* pellets and confluent monolayers were brought into the anaerobic chamber. *C. difficile* pellets were resuspended in pre-reduced serum-free DMEM+25 mM CaCl_2_ and placed on the monolayers to adhere for a total of 40 min at 37°C anaerobically. The inoculum was serially diluted and titered on BHIS with antibiotics as appropriate to calculate MOI. Following attachment, the supernatant was removed, and the monolayers were washed twice with pre-reduced PBS to remove non-adherent bacteria. Monolayers were resuspended in 1 ml PBS and scraped up to quantify attachment. Recovered *C. difficile* were serially diluted in PBS and plated on BHIS with antibiotics as appropriate to enumerate adherent levels of bacteria. The percent adherence was calculated as the ratio of recovered *C. difficile* to input *C. difficile*, multiplied by 100.

### Sporulation Assessments

To determine the kinetics of *C. difficile* sporulation, overnight cultures of *C. difficile* in BHIS were subcultured 1:50 into BHI and grown to an OD_600_ = 0.4. These cultures were used to inoculate 50 ml of BHI per time point analyzed. At each time point, cultures were harvested by centrifugation for 10 min at 3,200 × g, and the supernatant was removed. Cell pellets were washed once in 1 ml PBS, centrifuged, and resuspended in 1 ml PBS prior to heat-shock for 20 min at 60°C. Following heat shock to inactivate remaining vegetative cells, the remaining spores were serially diluted in PBS and plated on BHIS-Taurocholate plates. Plates were incubated anaerobically for 24 h at 37°C and enumerated. Sporulation efficiency was calculated as the percentage of spores obtained per total terminal vegetative cell count (Merrigan et al., 2013).

### Motility Assays

A quantitative assessment of C. *difficile* motility was performed as previously described (Baban et al., 2013). Briefly, overnight cultures of *C. difficile* grown in BHIS were diluted 1:50 in fresh BHI and grown to mid-exponential growth phase (OD_600_nm = 0.6) at 37°C in anaerobic conditions. Five μL of culture was then stab inoculated into the center of one well of a 6-well tissue culture plate filled with 20 ml of *C. difficile* motility agar (BHI+0.3% agar). Culture plates were wrapped in plastic wrap, placed into a container with damp paper towels to prevent evaporation, and grown anaerobically for 72 h at 37°C. Following incubation, the diameter of the swim radius was measured and averaged. Results represent the average of three independent experiments.

### Toxin Analyses

*C. difficile* toxin in the culture supernatant was detected by immunoblotting using monoclonal antibodies against both TcdA and TcdB. *C. difficile* cultures were grown in 35 ml quantities four times as indicated, and vegetative cells were removed by centrifugation at 4 °C for 10 min at 3,200 × g. Thirty ml of culture supernatant was harvested and concentrated 1:50 on a 100 kDa molecular weight cut-off column (EMD Millipore, Billerica, MA). Concentrated proteins were quantitated using a standard BCA assay (ThermoFisher, Waltham, MA), and 50 μg of protein was loaded onto 7.5% TGX acrylamide gels (BioRad, Hercules, CA) for electrophoresis. Proteins were transferred to nitrocellulose membranes (BioRad) and TcdA and TcdB were immunoblotted using specific monoclonal antisera (Abcam) at 1:5,000 dilution. Samples were detected using a secondary horseradish-peroxidase conjugated rabbit anti-mouse IgG antibody (BioRad) at 1:10,000 concentration. Antibodies were detected using FemtoWest Max Sensitivity substrate (ThermoFisher).

The Alere Wampole A/B Toxin ELISA kit (Alere, Atlanta GA) was utilized to determine relative levels of combined TcdA and TcdB in the supernatant of *C. difficile* culture. Strains were grown for 72 h in BHI broth, and the supernatant was harvested by centrifugation. Supernatants were diluted 1:10 and 1:100 in fresh BHI and processed for ELISA as per the manufacturer's instructions to determine the appropriate working concentration. Prior to analysis, the amount of total protein was normalized using the BCA assay (Pierce) to allow direct comparison of toxin levels among different *C. difficile* strains. Following assay processing as per the manufacturer's instructions, quantification of *C. difficile* toxin was performed by reading absorbance at 450 nm using an automated microtiter plate reader (Bio-Tek, Winooski VT).

### Statistical Methods

The Excel-Stat application was used for statistical analysis. Student's *t*-tests were used to compute differences between two groups, and error bars were calculated from standard deviation(s). ANOVA was used to compare >2 groups, followed by Tukey's honest significant difference (HSD) test for *post-hoc* analysis.

## Results

### *C. difficile* Encodes a Unique Sigma-54 Ortholog

**Figure 1:**
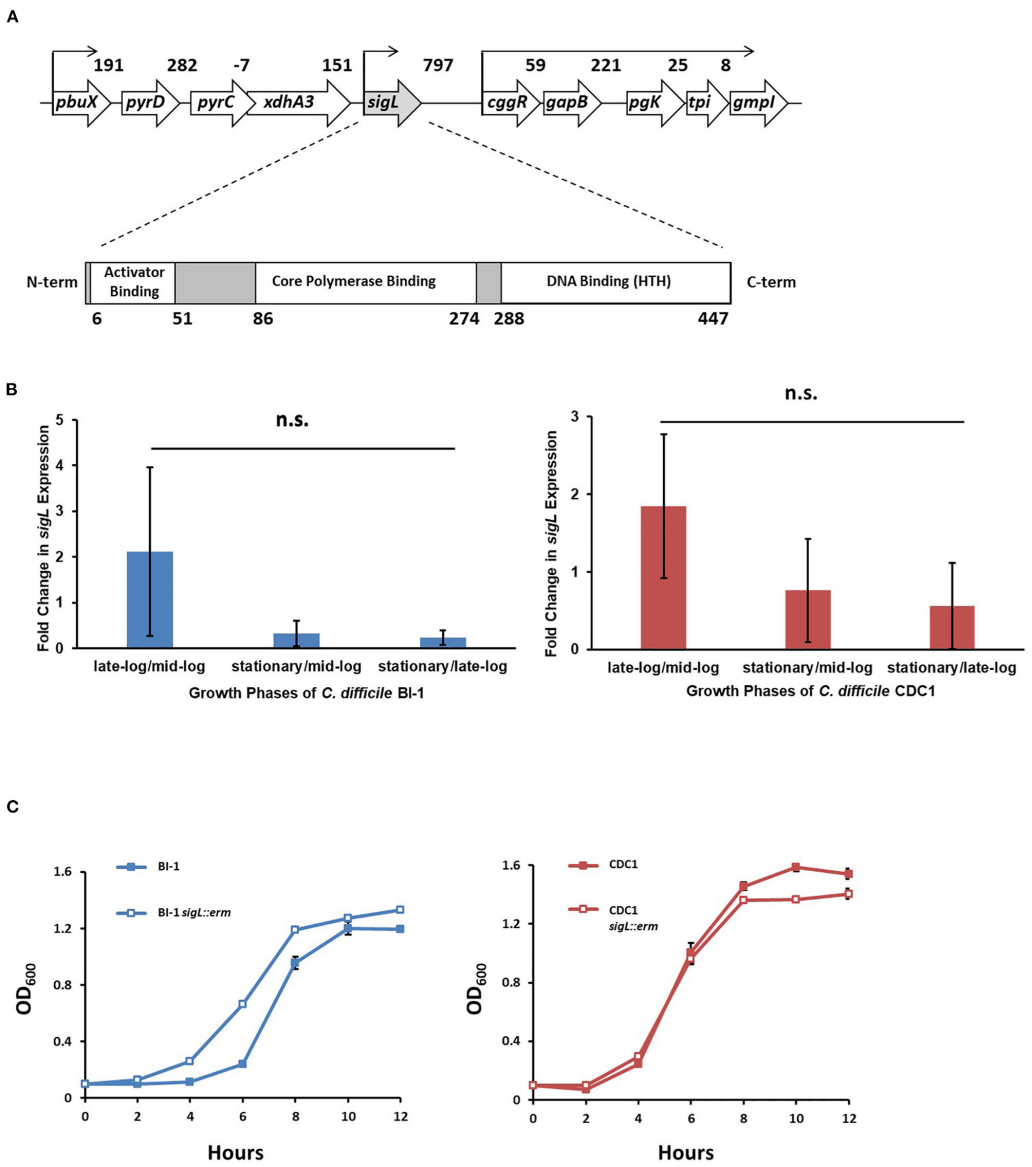
*sigL* loci, expression, and growth kinetics in *C. difficile* BI-1 and CDC1. **(A)** *C. difficile sigL* locus, showing predicted promoters, based on strain BI-1 genome sequence, and SigL conserved binding domains. Numbers in the upper schematic represent intergenic distances, and numbers below the lower schematic indicate amino acids. **(B)** Quantitative RT-PCR of *sigL* in BI-1 and CDC-1 across multiple growth phases. *16s* was used as a housekeeping control for qRT-PCR. Fold changes in expression represent the average of three biological replicates with standard deviation. n.s. = not significant **(C)** Growth of BI-1 and CDC1 strains compared to respective *sigL::erm* mutants. Cultures were grown in triplicate.

The *C. difficile* BI-1 (RT027) *sigL* ortholog is located immediately downstream from a putative purine biosynthesis locus (Figure 1A). The encoded 454 amino acid protein includes conserved sigma-54 DNA-, RNA polymerase-, and transcriptional activator-binding domains. *sigL* is conserved among all sequenced *C. difficile* genomes, and the predicted amino acid sequence is identical between BI-1 and CDC1 strains.

### *C. difficile sigL* Is Expressed in Multiple Growth Phases and Is Not Essential for Growth

During *C. difficile* growth, *sigL* expression was highly variable. While not significantly different between growth phases, expression trends showed the highest expression in the late-exponential phase, and lowest in early growth- and stationary phases. Importantly, the expression of *sigL* was not restricted to a specific phase of growth. These expression trends were similar in BI-1 and CDC1 strains (Figure 1B, Supplementary Figure S1). To interrogate the contribution of *sigL* to *C. difficile* biology, a lactococcal retrotransposition approach was used to generate isogenic mutants and corresponding complements in strains BI-1 and CDC1 (Kuehne and Minton, 2012) (Supplementary Figure S2). While the *sigL::erm* strains did not exhibit viability defects, they displayed modest, distinct, and reproducible differences in growth kinetics relative to the isogenic parent strains (Figure 1C).

### SigL Regulates the Abundance of Diverse Proteins

Soutourina and colleagues identified thirty SigL-dependent promoter sequences controlling 95 genes in the *C. difficile* strain in the laboratory-adapted 630Δ*erm* strain, using strain 630 as a reference genome. Twenty-four of the identified genes or operons were adjacent to enhancer-binding protein (EBP) genes involved in sigma-54-dependent activation (Soutourina et al., 2020). They also noted that transcription levels of genes or operons in 12 of the 30 putative *sigL* promoter sequences were downregulated in microarray analysis of the *C. difficile* 630Δ*erm sigL::erm* mutant (GEO accession GPL10556). We used the 12 putative *sigL* promoter sequences whose genes or operons were downregulated to generate a *sigL* positional weight matrix (PWM); this was used for promoter-proximal SigL binding site determination by employing the Find Individual Motif Occurrences (FIMO) tool (Grant et al., 2011).

Consistent with Soutourina et al. (2020), FIMO identified all 30 putative *sigL* promoter sites in both the 630 and BI-1 genomes, and notable differences from these two strains in the RT078 isolate, CDC1 (Table 1, Supplementary Table S1). First, only 28 of the 30 sites were conserved in CDC1; CD0166-CD0165 (peptidase, amino acid transporter) and CD2699-CD2697 (membrane protein, peptidase) operons and their respective *sigL* promoter sequences were either missing or disrupted relative to 630 and BI-1 (Table 1). Second, the CD0284-CD0289 operon (PTS Mannose/fructose/sorbose family) exhibited a substantially lower FIMO score compared to 630 and BI-1, with an atypical G residue at position 18 of the PWM. Similarly, CD3094 (sigma-54-dependent transcriptional regulator) has an atypical G residue at position 17 in the CDC1 promoter site (Table 1). Third, all three strains exhibited unique insertions/deletions in the *sigL*-dependent *prd* operon, linked to proline metabolism (Supplementary Figure S3). CD3246, a putative surface protein, is inserted between *prdC* and *prdR* in *C. difficile* 630, and is absent or truncated in BI-1 and CDC1. *prdB*, encoding a proline reductase, is present in 630 and BI-1 but truncated and missing a functional domain in CDC1 (Supplementary Figure S3). Of the previously identified EBPs for *sigL*-dependent promoters, all 24 identified in *C. difficile* 630 (Nie et al., 2019; Soutourina et al., 2020) were conserved in BI-1, while two EBPs (CD0167 and CD2700) were missing in CDC1 (data not shown).

**Table 1:** *sigL* positional weight matrix and *sigL* motif sequences with FIMO scores in *C. difficile* strains 630, BI-1, and CDC1.

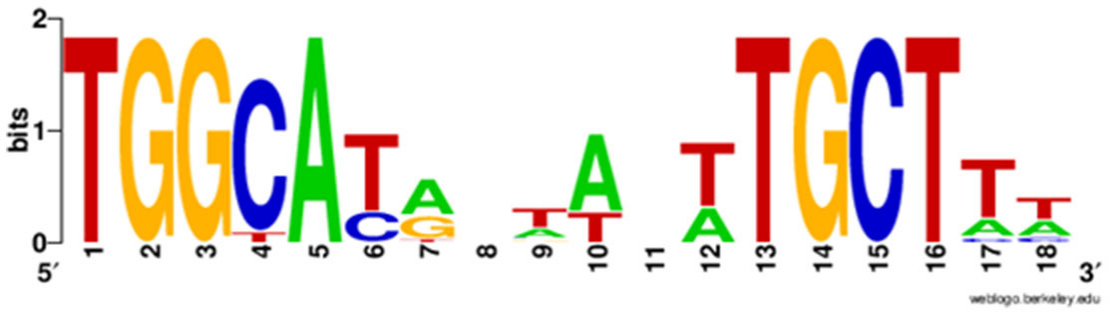						
**Operon (Soutorina et al.)**	***C. difficile*** **strain**	**Locus ID**	**Name**	**FIMO Score**	**FIMO** ***q*****-value**	**Motif sequence**
CD0166-0165	630	CD630_01650	putative amino acid transporter	26.888	0.001	TGGCATAATAATTGCTTA
	BI-1	CDBI1_00900	amino acid permease	26.888	0.001	TGGCATAATAATTGCTTA
	CDC1	**Not identified**				
CD2699-CD2697	630	CD630_26970	putative peptidase, M20D family	22.888	0.007	TGGCATAAGTTTTGCTTA
	BI-1	CDBI1_13130	amidohydrolase	22.888	0.007	TGGCATAAGTTTTGCTTA
	CDC1	**Not identified**				
CD0284-0289	630	CD630_02880	PTS system, mannose/fructose/sorbose IIC component	22.469	0.008	TGGCACGGCAATTGCTTA
	BI-1	CDBI1_01540	PTS sugar transporter subunit IIC	22.469	0.009	TGGCACGGCAATTGCTTA
	CDC1	CdifQCD-2_020200001377	PTS sugar transporter subunit IIC	**14.949**	0.091	TGGCACGGCAATTGCTT**G**
CD3094	630	CD630_30940	Transcriptional regulator, sigma-54-dependent	25.010	0.002	TGGCACAATTTTTGCTTT
	BI-1	CDBI1_14920	sigma 54-interacting transcriptional regulator	25.010	0.002	TGGCACAATTTTTGCTTT
	CDC1	CdifQCD-2_020200015331	putative sigma-54-dependent transcriptional regulator	**14.918**	0.091	TGGCACATTTTTTGCT**G**T
Not Identified	630	CD630_34780	conserved hypothetical protein	22.918	0.007	TGGCATGTTAGTTGCTTA
	BI-1	CDBI1_17010	EF2563 family selenium-dependent molybdenum hydroxylase system protein	22.918	0.007	TGGCATGTTAGTTGCTTA
	CDC1	CdifQCD-2_020200017431	Xanthine and CO dehydrogenases maturation factor, XdhC/CoxF family	22.918	0.008	TGGCATGTTAGTTGCTTA

*sigL-dependent operons identified by Soutorina et al. are listed in the first column, with the exception of CD630_34780 identified in our study (bottom row). Associated strains, loci, names, FIMO scores/q-values, and motif sequences are listed for the identified motifs. Two loci are not identified in CDC1 (column 3). Lower FIMO scores are bolded, and atypical nucleotides in the motif sequences are bolded*.

**Figure 2:**
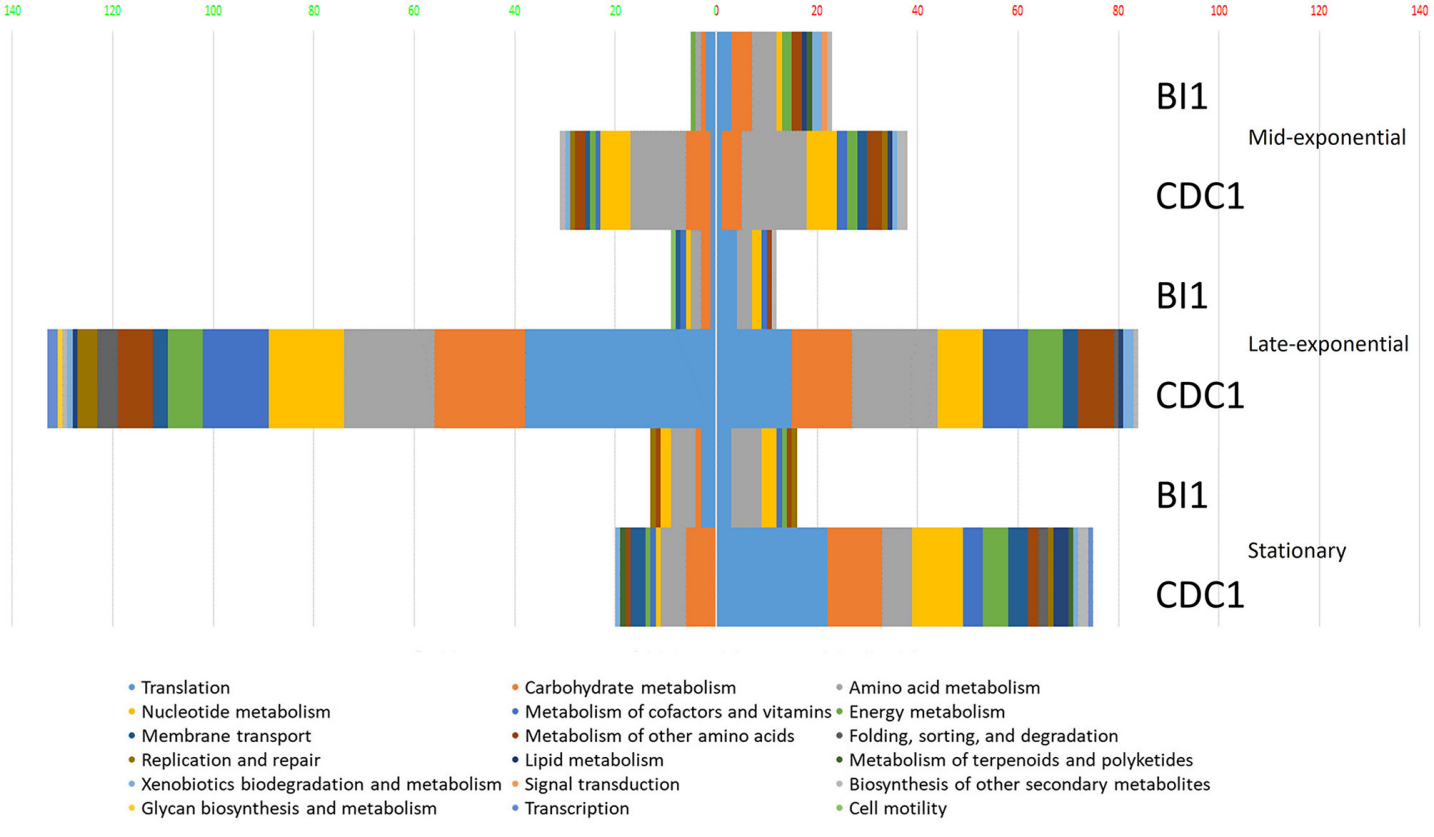
Functional classification of dysregulated proteins in *C. difficile* strains BI-1 and CDC1 resulting from *sigL* disruption. Functional classification analysis was performed on proteins identified as altered in *sigL* disruption mutants using an arbitrary fold change cut-off of 2. The x-axis is the number of dysregulated proteins. Numbers on the left in (green) are decreased in abundance, on the right (red) are increased in abundance. The colors of the columns represent different functional categories based on RAST classification system.

To gain a comprehensive appreciation of SigL-dependent regulation in ribotype RT027 and RT078 *C. difficile* strains, total protein abundances of BI-1, CDC1, and their respective *sigL::erm* derivatives were quantitated at mid-exponential (OD_600.6_ 0.6), late-exponential (OD_600.8_ 0.8), and stationary (OD_600_ 1) growth phases using quantitative mass spectrometry-based proteomics, and multiple stringent statistical tests. Overall, relative to the isogenic parent strains, more proteins were differentially abundant in CDC1 *sigL::erm* than in BI-1 *sigL::erm* across all three growth phases, but especially during late-exponential growth (Figure 2, Supplementary Table S2). For BI-1, 335 of 1426 identified proteins were altered in abundance in *sigL::erm* in at least one growth phase relative to WT. This corresponds to 24.9% of all expressed proteins for this strain. For CDC1, 516 proteins were altered in abundance in *sigL::erm*, corresponding to 73.5% of all expressed proteins for this strain. There was also a marked decrease in the abundance of proteins implicated in isoleucine fermentation and short-chain fatty acid metabolism in *sigL*::*erm* for both strains, and across all growth phases (Supplementary Table S2). Based on the markedly different protein profiles between the two strains, we further explored strain-specific SigL-dependent regulation of *C. difficile* physiology, including cell surface-related phenotypes, sporulation kinetics, biofilm formation, and toxin production.

### *sigL* Mutants Display an Altered Cell Surface

Relative to the BI-1 WT strain, *sigL::erm* had 47 surface-associated proteins with altered abundance (21 upregulated, 28 downregulated), and for CDC1, *sigL::erm* had 61 surface-associated proteins with altered abundance (44 upregulated, 34 downregulated) (Supplementary Table S3). Proteomics findings also revealed that dysregulated molecules included those predicted to be involved in S-layer display, host-cell adhesion, antimicrobial peptide resistance, and transport (Supplementary Table S3). Therefore, we probed the surface properties of the *sigL::erm* mutants relative to the respective isogenic parent strains.

**Figure 3:**
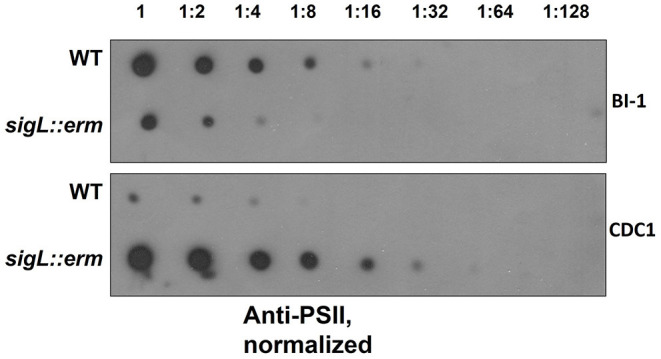
PSII abundance in *sigL::erm* differs with respect to wild-type strain BI-1 and CDC1. Serially-diluted cell extracts of WT and *sigL::erm* derivatives (normalized to OD600 = 1.2 in BHI) were spotted on PVDF membranes and immunoblotted using PSII-specific antiserum.

PSII, an antigenically-important surface polysaccharide unique to *C. difficile* strains, is hypothesized to assemble on the inner face of the cell membrane and translocated to the cell surface *via* the action of a flippase, MviN (Willing et al., 2015; Chu et al., 2016). Perturbations in PSII biogenesis and/or cell surface display result in diminished extractability of this molecule (Chu et al., 2016). Our proteomics findings revealed alterations in abundance of cell wall proteins Cwp2 and SlpA, to which PSII binds (Willing et al., 2015). Immunoblotting revealed diminished surface-extractable PSII in BI-1 *sigL::erm* compared to the isogenic parent strain. In contrast, however, PSII was more readily released from CDC1 *sigL::erm* strain relative to the isogenic parent (Figure 3). Thus, SigL influences expression, export and/or assembly of key *C. difficile* surface molecules in a strain-specific manner.

**Figure 4:**
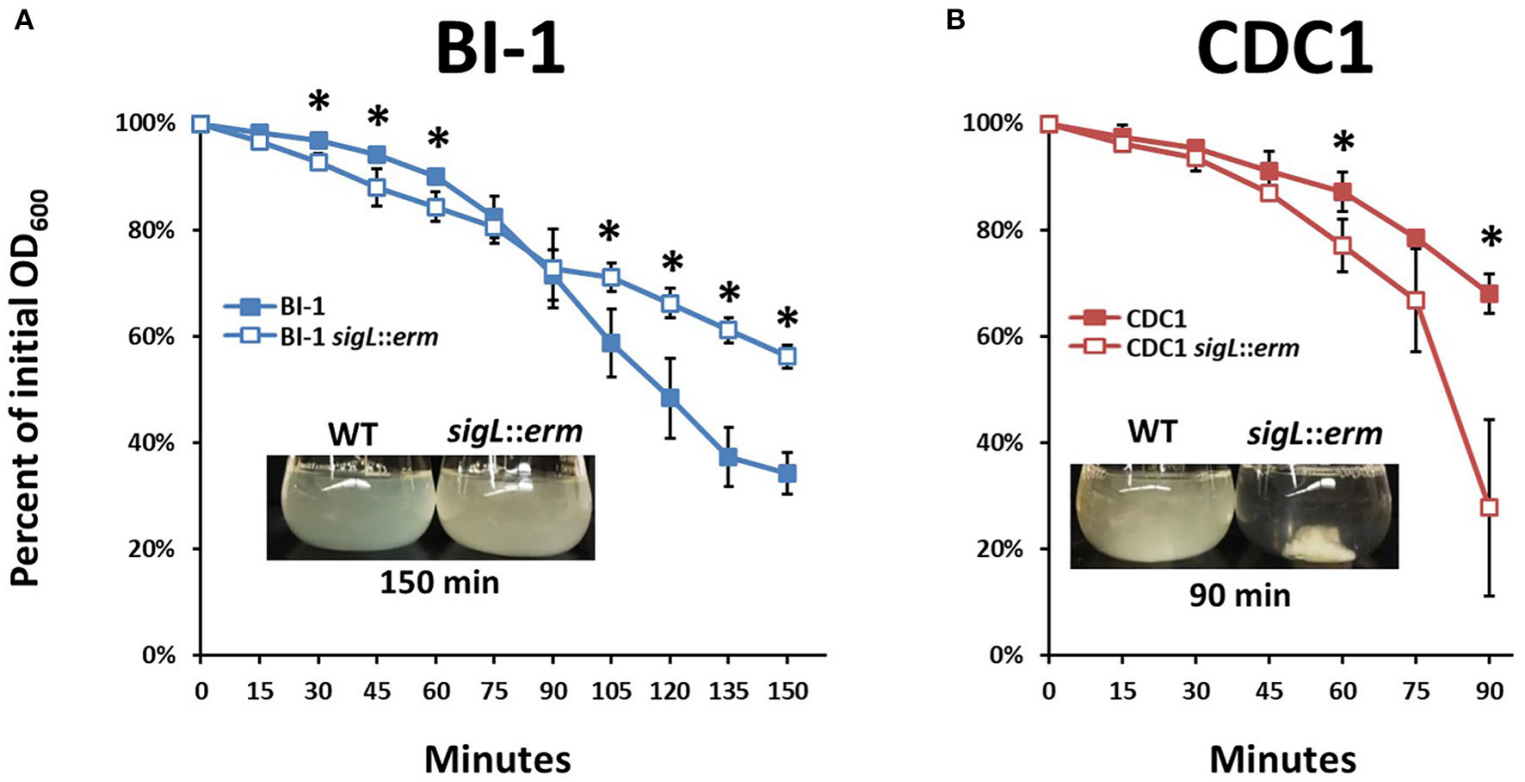
Autolysis rates of *C. difficile* strains and *sigL* mutants differ between BI-1 and CDC1. Cultures were grown to OD_600_ level of approximately 1.2, and OD_600_ values were measured as percent of initial OD over time following the addition of Triton X-100. Visualization of autolysis in **(A)**. BI-1 and **(B)**. CDC1 compared to respective *sigL::erm* mutants are show as insets. Each assay was performed in biological triplicate. ^*^p < 0.05.

Changes in bacterial surface architecture impact autolysis susceptibility; this has implications for toxin release in *C. difficile* (Tsuchido et al., 1990; Wydau-Dematteis et al., 2018; Mayer et al., 2019). Surface composition dictates the ease with which detergents, such as Triton X-100, interact with lipoteichoic acid from Gram-positive bacterial cell walls, which in turn influences the activation of autolytic enzymes (Komatsuzawa et al., 1994; Fujimoto and Bayles, 1998). To establish the influence of SigL on *C. difficile* cell surface properties, we quantitatively measured rates of autolysis between wild type and *sigL::erm* mutants of both BI-1 and CDC1. While autolysis rates were similar between the BI-1 WT and *sigL::erm* strains at early time points, the mutant demonstrated decreased autolysis at later time points. In contrast, the CDC1 *sigL::erm* strain exhibited a more rapid rate of autolysis than the WT strain, and underwent complete autolysis in 90 min (Figures 4A,B).

### Loss of SigL Perturbs *C. difficile* Aggregation in a Strain-Specific Manner

**Figure 5:**
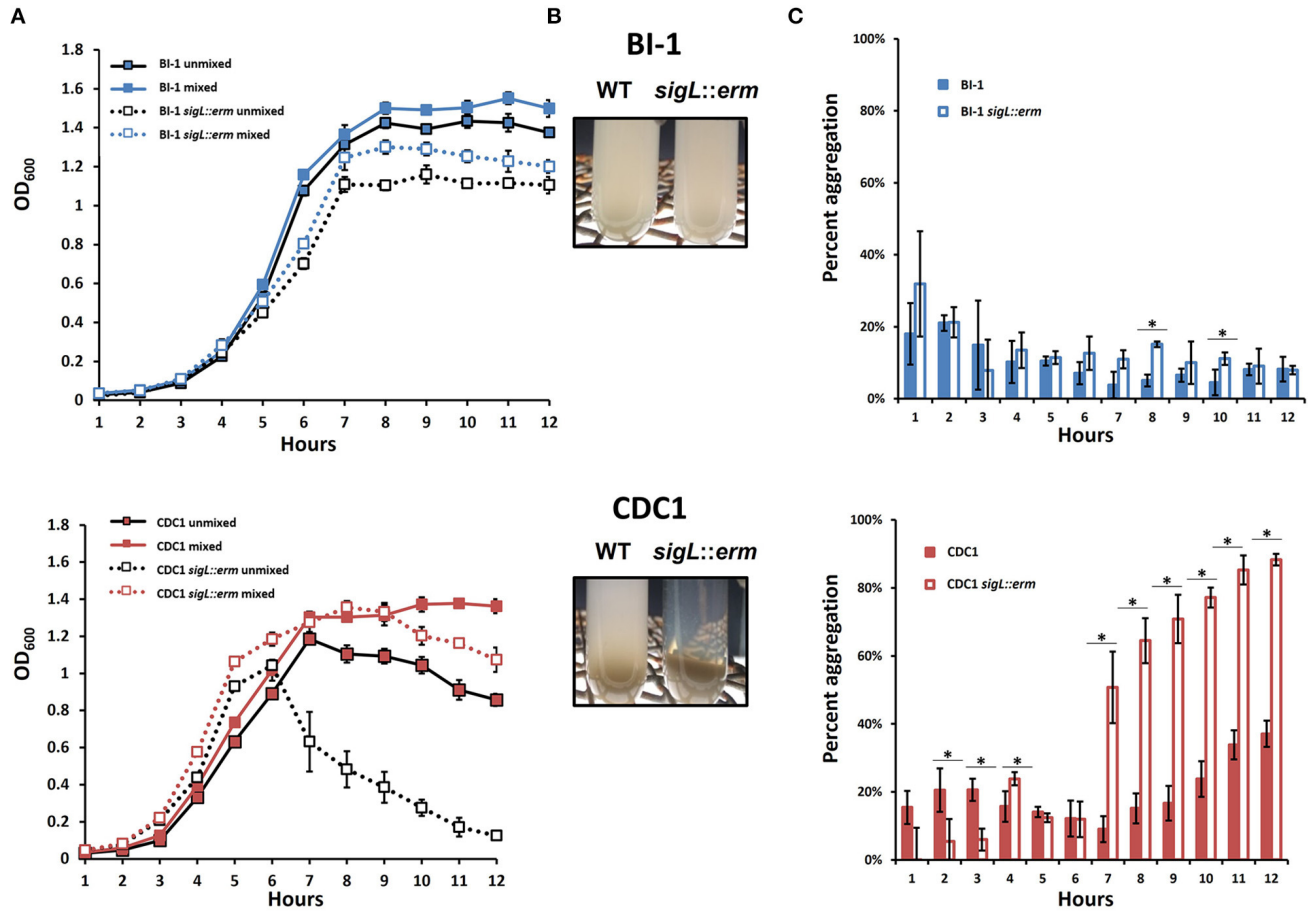
Aggregation of *C. difficile* strains and respective *sigL::erm* mutants differs between BI-1 and CDC1. **(A)** OD_600_ values of the top 1 ml of culture (unmixed) and a replicate vortexed culture sample (mixed) were read hourly to determine sedimentation curves. **(B)** Visualization of aggregation in BI-1 (top) and CDC1 (bottom) WT and *sigL::erm* strains.**(C)** Percent aggregation of *C. difficile* strains and respective *sigL::erm* mutants, calculated as the difference in OD_600_ values between the whole (mixed) culture and top 1 ml (unmixed) culture, as a percentage of the mixed culture OD_600_ value. Cultures were grown in biological triplicate. WT and *sigL::erm* pairs were compared at each time point using Student's *t*-test. ^*^p < 0.05.

Changes in cell surface protein composition, including variation in charge and hydrophobicity, can dictate bacterial aggregation which, in turn, can influence virulence (Misawa and Blaser, 2000). Therefore, we monitored aggregation rates of both BI-1 and CDC1 *sigL::erm* mutants during growth in Brain Heart Infusion (BHI). While the aggregation rate of *C. difficile* BI-1 and the isogenic *sigL::erm* remained unchanged throughout growth, CDC1 *sigL::erm* was more aggregative than the parent WT strain during late-exponential and stationary growth phases (Figures 5A,B). These phenotypes were independently verified *via* autoagglutination assays using plate-grown cultures. Again, CDC1 *sigL::erm* agglutinated significantly more than the cognate wildtype strain at multiple time points (Figure 5C). Autoagglutination rates of the BI-1 *sigL::erm* were not different from wild type with the exception of two time points at 8 and 10 h (Figure 5C).

### *C. difficile sigL* Mutants Exhibit Altered Propensity to Form Biofilms

**Figure 6:**
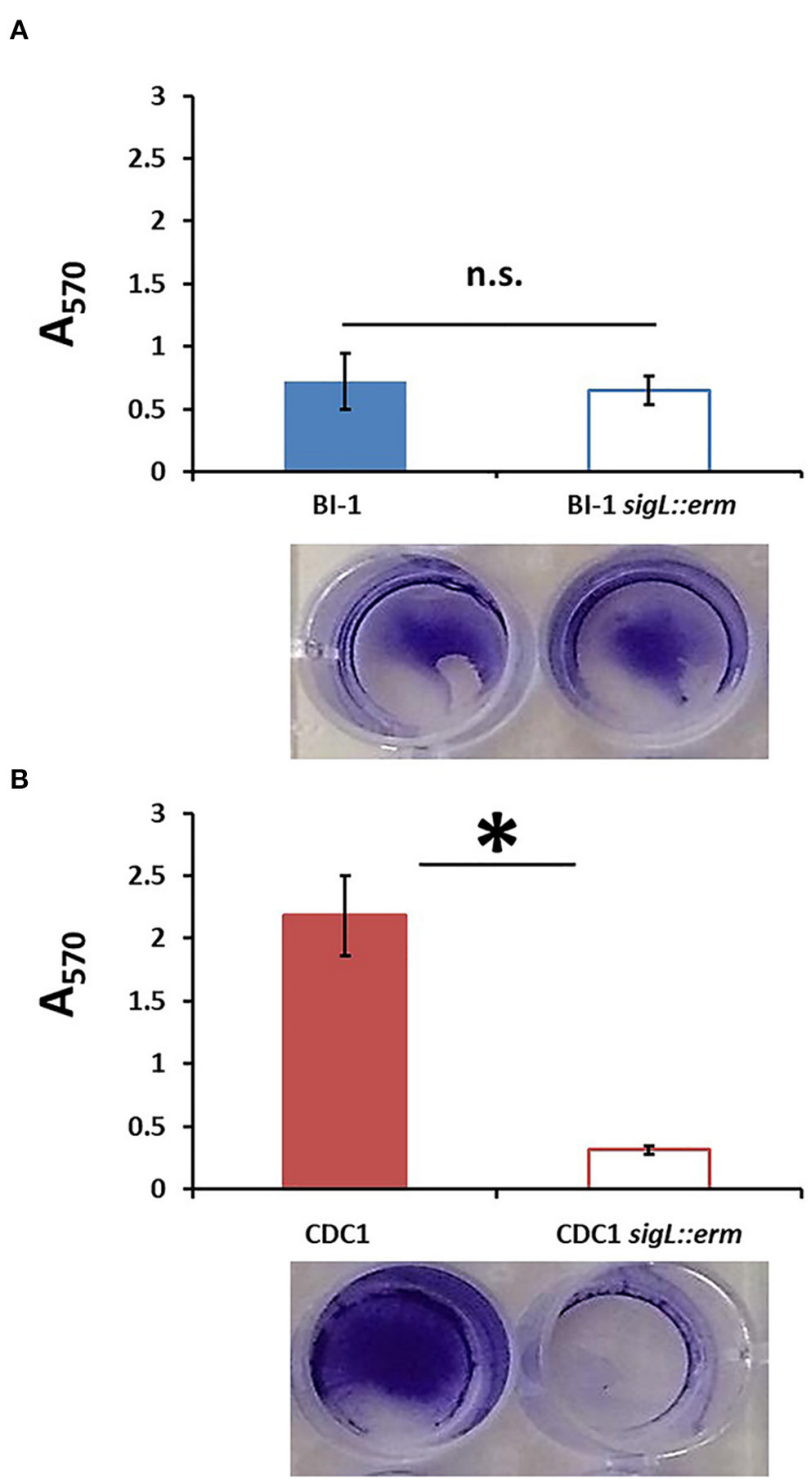
Biofilm formation is reduced in CDC1 *sigL::erm*, but not in BI-1 *sigL::erm*. Biofilm was quantified by the release of crystal violet retained by the *C. difficile* biomass after 72 h. Absorbance was measured at 570 nm. **(A)** Biofilm formation in BI-1 and BI-1 *sigL::erm*. **(B)** Biofilm formation in CDC1 and CDC1 *sigL::erm*. Samples were grown in biological triplicate and compared using Student's *t*-test. ^*^p < 0.05.

Altered surface properties can influence the ability to form biofilms; in *C. difficile*, this can impact pathogen persistence in the gut (Pantaléon et al., 2015; Fernández Ramírez et al., 2018; Chamarande et al., 2021; JaneŽ et al., 2021; Kumari and Kaur, 2021). Disruption of SigL had no discernable impact on the ability of BI-1 strains to form biofilms grown in liquid culture on polystyrene plates (Figure 6A). In contrast, CDC1 *sigL::erm* had decreased biofilm density relative to the isogenic parent strain (Figure 6B). Complementation restored the CDC1 phenotype (Supplementary Figure S4A), and overexpression of SigL enhanced the ability of the CDC1 strain, but not BI-1, to form a biofilm (Supplementary Figures S4B,C).

### *sigL* Mutants Exhibit Altered Levels of Attachment to Cultured Human Colonic Intestinal Epithelial Cells

**Figure 7:**
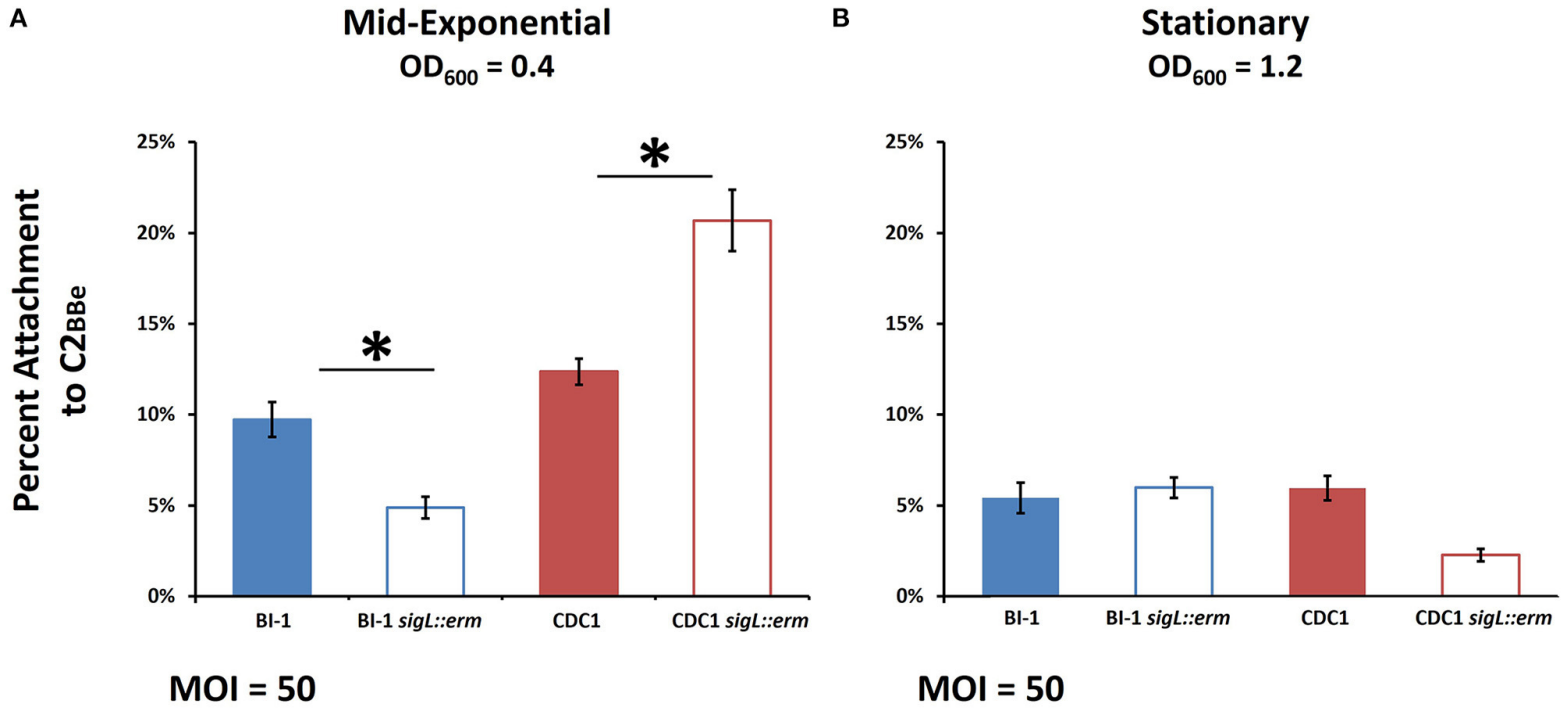
Host cell attachment in *sigL::erm* is phase- and ribotype-dependent. *C. difficile* cultures were grown to mid-exponential or stationary phase and added to Caco-2_BBe_ intestinal epithelial cell monolayers. Percent attachment was calculated by adhered CFUs as a percentage of the initial inoculum CFUs. **(A)** Attachment to Caco-2_BBe_ intestinal epithelial cells in mid-exponential phase. **(B)** Attachment to Caco-2_BBe_ intestinal epithelial cells in stationary phase. The assays were conducted in biological triplicate and samples were compared using ANOVA and Tukey's HSD *post-hoc* test. ^*^p < 0.05.

Variations in cell surface composition leading to alterations in phenotypic properties, including aggregation, can affect bacterial attachment to host cells (Burdman et al., 2000; Merrigan et al., 2013; Foster, 2019), and this has profound implications for *C. difficile* host establishment (Vedantam et al., 2018). We, therefore, compared the adherence of parental and *sigL* mutant strains to Caco-2_BBe_ intestinal epithelial cells. Two different growth phases were chosen for analysis representing differential protein expression in *sigL::erm* strains (Figure 7): OD_600_ = 0.4, mid-exponential, and OD_600_ = 1.2, stationary. For mid-exponential phase bacteria, compared to the respective parent strains, BI-1 *sigL::erm* exhibited diminished levels of attachment, whereas CDC1 *sigL::erm* attachment was more robust (Figure 7A). For bacteria in the stationary phase, there was no apparent difference in attachment between the WT and the mutant in the BI-1 background, but CDC1 *sigL::erm* was less adherent to host cells relative to the isogenic WT strain (Figure 7B). Thus, the contribution of SigL toward the ability of *C. difficile* to attach to biotic surfaces is both strain- and growth phase-dependent.

### Loss of *sigL* Alters Sporulation Kinetics in an RT078 Background

**Figure 8:**
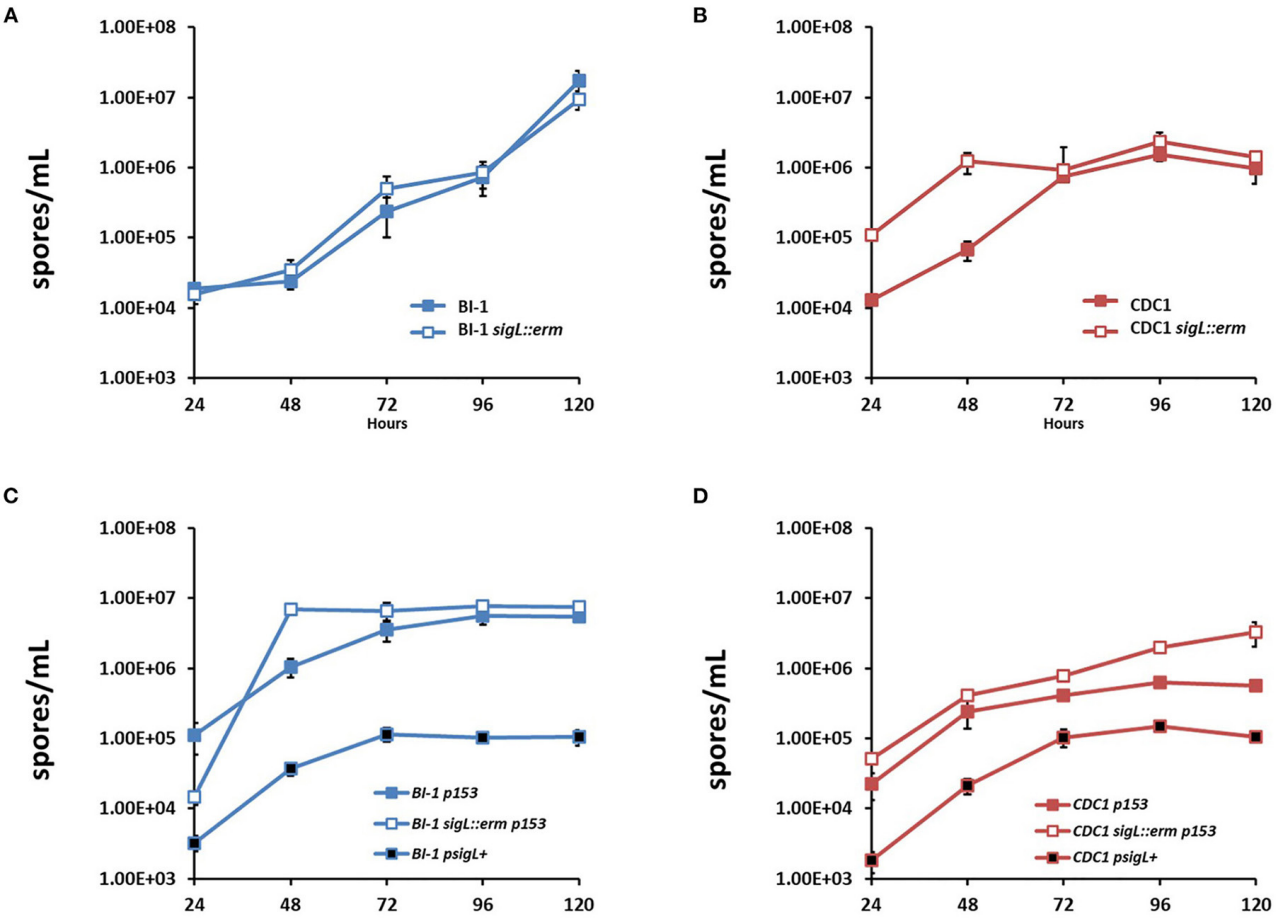
Sporulation kinetics are altered in *sigL::erm* in a ribotype-dependent manner. Spores were enumerated by heat shocking and plating cultures on BHIS-Taurocholate at each time point. For overexpression, WT *C. difficile* strains were conjugated with empty vector pMTL82153 (p153). *sigL::erm* in BI-1 and CDC1 backgrounds were conjugated with empty vector or p153 expressing *sigL* under a constitutive promoter (psigL+). **(A)** Spore enumeration over 120 h in BI-1 and BI-1 *sigL::erm* strains. **(B)** Spore enumeration over 120 h in CDC1 and CDC1 *sigL::erm* strains. Cultures were grown in biological triplicate. **(C)** Spore enumeration over 120 h in BI-1 *sigL* overexpression strains. **(D)** Spore enumeration over 120 h in CDC1 *sigL* overexpression strains. Cultures were grown in biological triplicate.

*C. difficile* sporulates in response to a combination of unfavorable extracellular environment and nutritional/metabolic deprivation, and sporulation efficiency can impact bacterial gut persistence (Paredes-Sabja et al., 2014; Shen et al., 2019). We next assessed if loss of SigL influences *C. difficile* sporulation kinetics, by measuring the outgrowth of preformed spores following the heat shock of vegetative cells BI-1 and BI-1-*sigL::erm* had comparable spore titer levels over a period of 5 days, indicating that SigL does not influence sporulation processes in this strain (Figure 8A). In contrast, CDC1 *sigL::erm* had higher spore titers than the WT strain at 24 and 48 h, but the difference resolved at later time points (72–120 h) (Figure 8B). Sporulation efficiency was also calculated in our *C. difficile* strains and respective *sigL::erm* derivatives, with spore titers as a percent of terminal vegetative cell titers. These data show higher sporulation efficiencies at earlier time points in the CDC1 *sigL::erm* strain (Supplementary Figure S5), commensurate with the higher spore titers observed in Figure 8B; these also demonstrate a SigL-dependent effect on sporulation efficiency in CDC1 that is absent in BI-1 (Supplementary Figure S5). Plasmid-driven SigL overexpression, using pMTL82153, which expresses *sigL* under a constitutive promoter, reduced sporulation of both the CDC1- and BI-1-*sigL::erm* mutants, as well as the corresponding WT strains (Figures 8C,D).

We next assessed if the increased sporulation phenotype of CDC1 *sigL::erm* could be reversed by plasmid-complementation, using pMTL82151, which expresses *sigL* under a native promoter. Plasmid (empty vector)-harboring WT- and mutant strains recapitulated the sporulation phenotypes for CDC1 though differences in overall kinetics were observed (likely due to the presence of the antibiotic used for plasmid maintenance). This was reversed by a *sigL-*encoding plasmid (Supplementary Figure S6). Spore production was also reduced in BI-1 with plasmid complementation.

### *C. difficile* BI-1 Motility Is Not Altered by *sigL* Inactivation

Sigma-54 is widely recognized as an important regulator of flagellar biosynthesis in many Gram-negative species, but its role is non-uniform among different families of organisms (Tsang and Hoover, 2014). To evaluate the role of SigL in the regulation of flagellar synthesis in *C. difficile*, motility assays were performed using the motile strain BI-1. CDC1 is aflagellate due to the loss of a substantial part of the flagellar locus encoding early structural flagellar genes (Stabler et al., 2009), a hallmark of the RT078 ribotype, and was used as a negative control. In contrast to other bacterial systems whereby motility is influenced by sigma-54 expression, the motility and flagellar expression of *C. difficile* BI-1 were not significantly affected by *sigL* inactivation (Supplementary Figure S7).

### *C. difficile* Supernatant Toxin Levels Are Altered in *sigL* Mutants

**Figure 9:**
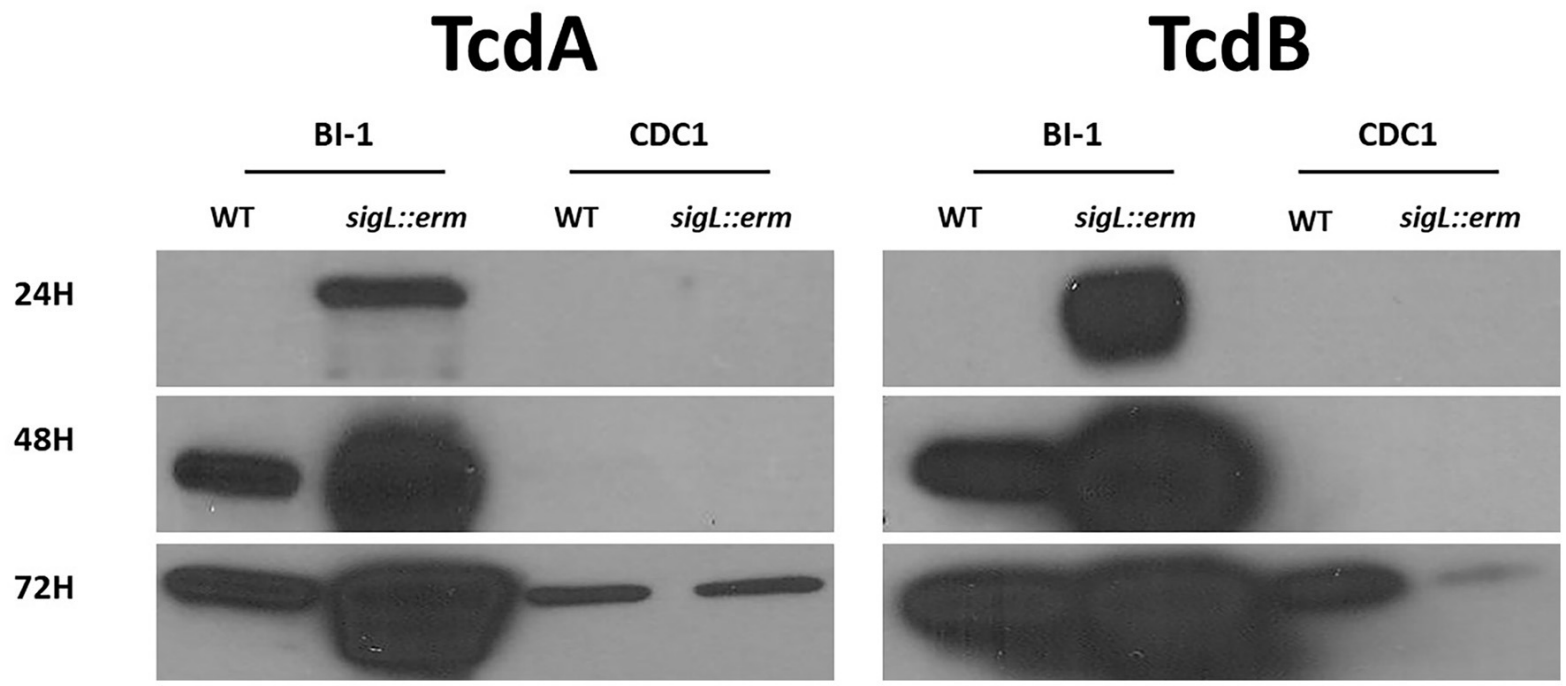
Supernatant toxin level is differentially altered in *sigL::erm* in BI-1 and CDC1 backgrounds. BI-1 and CDC1 *C. difficile* strains and respective *sigL::erm* mutants were grown for 24, 48, and 72 h. Vegetative cells were removed by centrifugation and the supernatant was assayed for toxins by immunoblotting using monoclonal antibodies against TcdA and TcdB. Blots are representative of three experiments.

*C. difficile* toxin expression is influenced by both metabolic factors and genetic determinants (Bouillaut et al., 2015; Martin-Verstraete et al., 2016; Ransom et al., 2018). Given the wide-ranging impact of SigL presence on *C. difficile* metabolic enzymes, we used immunoblot analysis to monitor TcdA and TcdB in WT and mutant culture supernatants from 24 to 72 h. Loss of SigL dramatically increased supernatant TcdA and TcdB levels in BI-1 but decreased the levels of the two toxins in the CDC1 background (Figure 9). This was confirmed by the toxin ELISA (Supplementary Figure S8). Correspondingly, complementation decreased supernatant toxin levels by BI-1 *sigL::erm*, but increased TcdA and TcdB levels in the CDC1 background (Supplementary Figure S8).

## Discussion

Although rarely an essential protein, no unified functional theme has been identified concerning the sigma-54 dependent metabolic repertoire. This is in contrast with the sigma-70 counterpart, which often serves dedicated functions. Sigma-54-dependent proteins have pleiotropic functions in bacterial systems, with roles ranging from carbohydrate and nitrogen metabolism to modification of cell surface structures, including flagella and pili (Alm et al., 1993; Francke et al., 2011; Hayrapetyan et al., 2015). In Gram-positive organisms, the Sigma-54, SigL, regulates adaptation to environmental conditions, including cold shock (Wiegeshoff et al., 2006), osmotic stress (Okada et al., 2006), and oxidative stress (Stevens et al., 2010), and is involved in amino acid catabolism (Debarbouille et al., 1999), acetoin cycle regulation (Ali et al., 2001; Francke et al., 2011), and flagellar synthesis (Hayrapetyan et al., 2015).

A previous report revealed that *C. difficile sigL* transcript levels were highest during the stationary phase of growth (Karlsson et al., 2008). In this study, we observed a trend of higher SigL expression during the late-exponential phase, though the expression was present and highly variable during all phases of growth, indicative of the biological relevance of SigL throughout the *C. difficile* life cycle. Due to its requirement for activation prior to transcription, it is likely *C. difficile* SigL function is regulated through the coordinated expression of EBPs, driving SigL transcription of target operons when physiologically necessary. The number of EBPs roughly correlates with genome size, with *C. difficile* strains 630 and BI-1 harboring 23 such genes, two of which are absent in CDC1 (Nie et al., 2019).

Promoters recognized by members of the sigma-54 family have highly conserved −24 and −12 regions upstream of the transcriptional start site, including a GC dinucleotide at position −12 and GG at position −24 (Barrios et al., 1999; Zhang and Buck, 2015). Barrios et al. identified 85 SigL promoter sequences in multiple bacterial species, with the consensus sequence “TTGGCATNNNNNTTGCT” used to inform more recent studies to search for promoter candidates in *Clostridiales* genomes (Barrios et al., 1999; Nie et al., 2019). While *C. difficile* SigL interaction with specific promoters was described previously (Nie et al., 2019; Soutourina et al., 2020), the relative strength of the promoters, or the essentiality of specific residues in the promoter for interaction, has not been defined.

We identified SigL-dependent promoter sequences in BI-1 and CDC1 strains in agreement with previous studies in *C. difficile* 630Δ*erm*, using strain 630 as a reference strain, with SigL-dependent regulation of genes involved in amino acid metabolism and sugar transport, in addition to novel promoter sequences not identified in previous 630 studies (Soutourina et al., 2020). In BI-1, a promoter sequence for an EF2563 family selenium-dependent molybdenum hydroxylase (CD630_3478/CDBI1_17010) was uniquely identified. This same EF2563 protein was identified in CDC-1 (CdifQCD-2_020200017431) (Table 1, Supplementary Table S1). Other strain-specific differences noted in the repertoire of SigL regulated genes included the absence of two SigL-dependent promoters in CDC1 compared to BI-1 and 630, corresponding to an amino acid transporter and a membrane protein/peptidase, respectively. Additionally, a G residue replaces the typical A or T at position 18 in the positional weight matrix (PWN) for the CD0284-CD0289 operon (PTS Mannose/fructose/sorbose family) and position 17 for the CD3094 operon, resulting in a lower FIMO score suggestive of decreased SigL interaction. These unique promoter features noted for BI-1 and CDC-1 extended to other published sequences of strains belonging to the RT027 and RT078 lineages, respectively.

In our proteomics analysis of BI-1 and CDC1, we observed profoundly pleiotropic, strain-specific, and growth-phase-specific SigL-dependent regulation. In the late-exponential phase, 224 proteins were downregulated in the CDC1*::erm* strain, compared to only 76 downregulated proteins in the BI-1 background for the same growth phase (Supplementary Table S2). Our proteomics data showed that several hydroxyisocaproyl-CoA dehydratase (Had) proteins, associated with L-leucine fermentation, were downregulated in *sigL::erm* in both strain backgrounds and across all growth phases, consistent with established roles for sigma-54 in amino acid fermentation (Gardan et al., 1995, 1997; Debarbouille et al., 1999). Our data also show that SigL-dependent protein regulation is not only highly pleiotropic within a strain, but widely differential between *C. difficile* 027 and 078 strains.

The SigL regulon is remarkably distinct between BI-1 (RT027) and CDC1 (RT078), and this is manifested *via* distinct phenotypes. Such differences have been shown for *C. difficile* strains R20291 (RT027) and 630Δ*erm* (RT012) specifically in the context of flagellar protein mutants (Baban et al., 2013), but comparative studies for sigma-54 mutants are lacking. A recent large-scale study identified evolutionary relationships between sigma-54 genes and the synthesis and transport of elements of the bacterial cell surface and exterior, including exopolysaccharides, lipids, lipoproteins, and peptidoglycan (Francke et al., 2011). In agreement, several dysregulated protein targets observed between the *C. difficile sigL* mutants were either components of, or associated with, the cell surface indicating dynamic compositional regulation by SigL. Phenotypic analysis of the *sigL* mutants of the two ribotypes confirms strain-specific surface alterations. Differential cell surface phenotypes with respect to aggregation, attachment to biotic surfaces, exopolysaccharide levels, and autolysis were observed.

Metabolic events and environmental conditions encountered during the stationary phase determine whether *C. difficile* vegetative cells will form biofilms, produce toxins and/or initiate sporulation. In this work, a strong correlation was observed between the expression of SigL, sporulation, biofilm, and toxin phenotypes. In CDC1, there is an inverse association between sporulation and toxin production: in CDC1 *sigL::erm*, spore titers and sporulation efficiency are increased, and biofilm and toxin accumulation in the supernatant are decreased relative to the parent strain. In BI-1, the relationship appears more complex. Sporulation and biofilm formation are not influenced by the loss of *sigL* expression in the BI-1 background; therefore, the reason for the accompanying increase in supernatant toxin in the BI-1 *sigL* mutant remains unknown, but our observed strain-specific differences in SigL-dependent operons may yield some insight. The *prd* locus is associated with proline metabolism, which along with other amino acids can be metabolized by *C. difficile via* Stickland reactions. This amino acid metabolism is associated with reduced toxin production (Bouillaut et al., 2013). We identified a SigL-dependent promoter for the *prd* locus; therefore, a contributing factor to the increased toxin in BI-1 *sigL::erm* may be differences in SigL-dependent proline metabolism. Protein levels in members of the *prd* locus, including the proline reductase PrdA and proline racemase PrdF, were shown to be similarly decreased in *sigL::erm* in both the BI-1 and CDC1 backgrounds (Supplementary Table S2), but interestingly, we observed a difference in the architecture of the *prd* locus in CDC1 compared to BI-1, with a truncation in *prdB* (Supplementary Figure S3). How this truncation may impact proline metabolism and toxin production in CDC1 requires further investigation. There are also potential epistatic effects of other regulators, such as SigD, that may affect toxin production.

A recent study in *C. difficile* 630Δ*erm* showed major proteomic differences in two models of biofilm-like growth: aggregate biofilms and colony biofilms, and demonstrated that SigL was significantly induced in aggregate biofilms (Brauer et al., 2021). The proteomic and phenotypic differences in our respective 027 and 078 *sigL::erm* mutants may be partially explained by strain-specific differences in the ability to aggregate and form biofilms, resulting in differential SigL induction. Indeed, WT BI-1 shows reduced baseline biofilm formation compared to WT CDC1 (Figure 6).

It is well-appreciated that strain-level variations may influence *C. difficile* virulence and epidemiology in fundamental ways, but less is known about the regulatory networks underlying these differences. Our studies suggest that the conserved regulator SigL has profoundly differential impacts on gene expression, including toxin production, in the predominantly human- and veterinary-associated *C. difficile* lineages RT027 and RT078, respectively. Future studies, including animal experiments, will be instructive in determining the consequences of these regulatory differences to disease establishment, progression, asymptomatic carriage, recurrence, and/or response to treatment.

## Data Availability Statement

The datasets presented in this study can be found in online repositories. The names of the repository/repositories and accession number(s) can be found in the article/Supplementary Material.

## Author Contributions

CA, AC, and KC performed the experiments. AC and KC analyzed the data and drafted the manuscript. BR performed most of the informatic analyses. VV and GV designed the study, obtained research funding, and supervised all aspects of the study. GV and VV contributed equally as senior authors to this study. All authors approved the submitted version of the manuscript.

## Funding

This work was supported by funding from the National Institutes of Health [R33AI121590531 (GV)] and the US Department of Veterans Affairs [Research Career Scientist Award IK6BX003789 (GV) and Merit Award I01BX001183 (GV)].

## Conflict of Interest

The authors declare that the research was conducted in the absence of any commercial or financial relationships that could be construed as a potential conflict of interest.

## Publisher's Note

All claims expressed in this article are solely those of the authors and do not necessarily represent those of their affiliated organizations, or those of the publisher, the editors and the reviewers. Any product that may be evaluated in this article, or claim that may be made by its manufacturer, is not guaranteed or endorsed by the publisher.
