# Supplementary figures and images for: The Alternative Sigma Factor SigL Influences Clostridioides difficile Toxin Production, Sporulation, and Cell Surface Properties
Source: Front Microbiol. 2022 May 11;13:871152. doi: 10.3389/fmicb.2022.871152 (PMC9130780; doi:10.3389/fmicb.2022.871152)

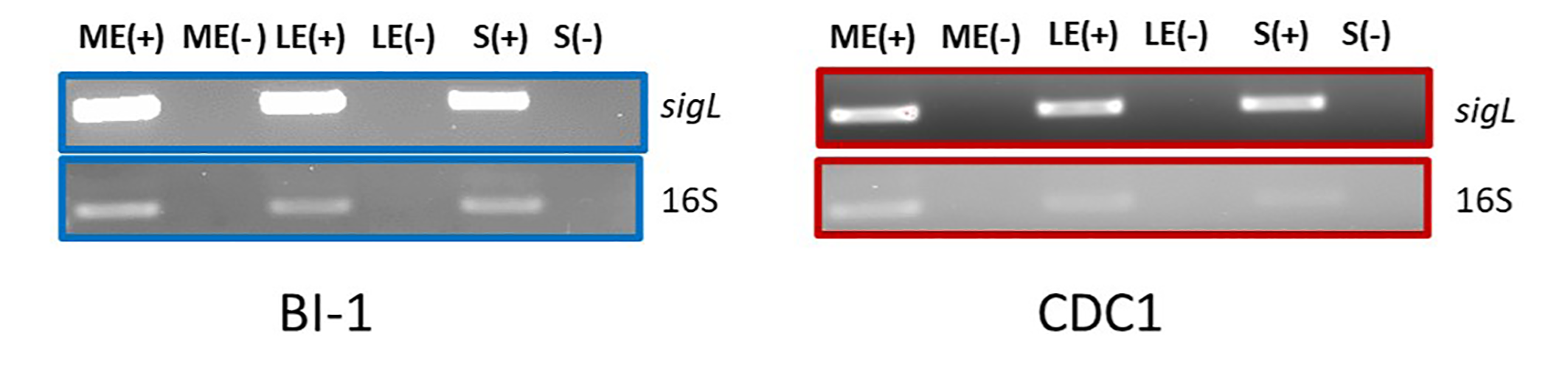

Supplement: Supplementary file 1 [file Image_1.TIF]

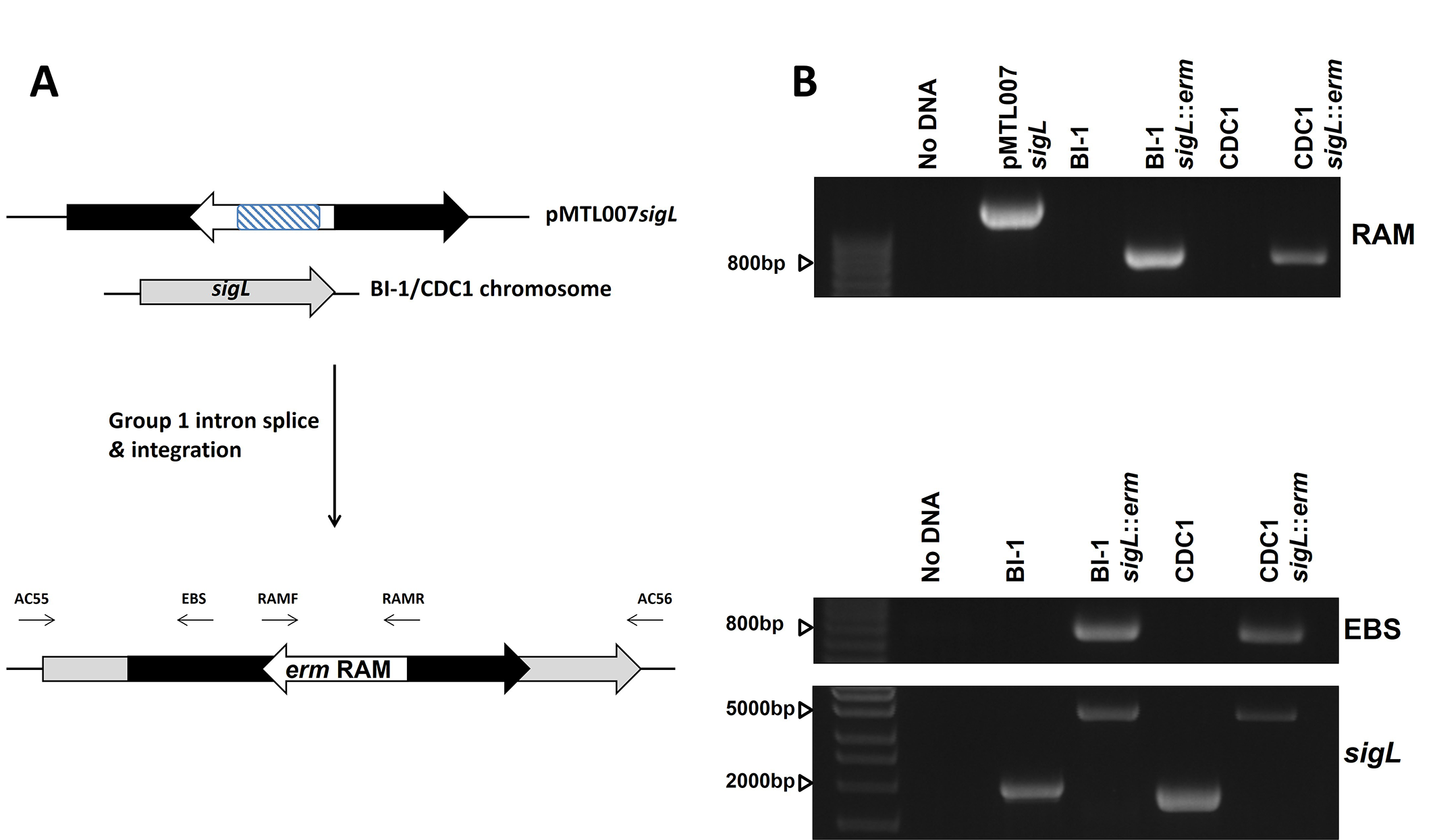

Supplement: Supplementary file 2 [file Image_2.TIF]

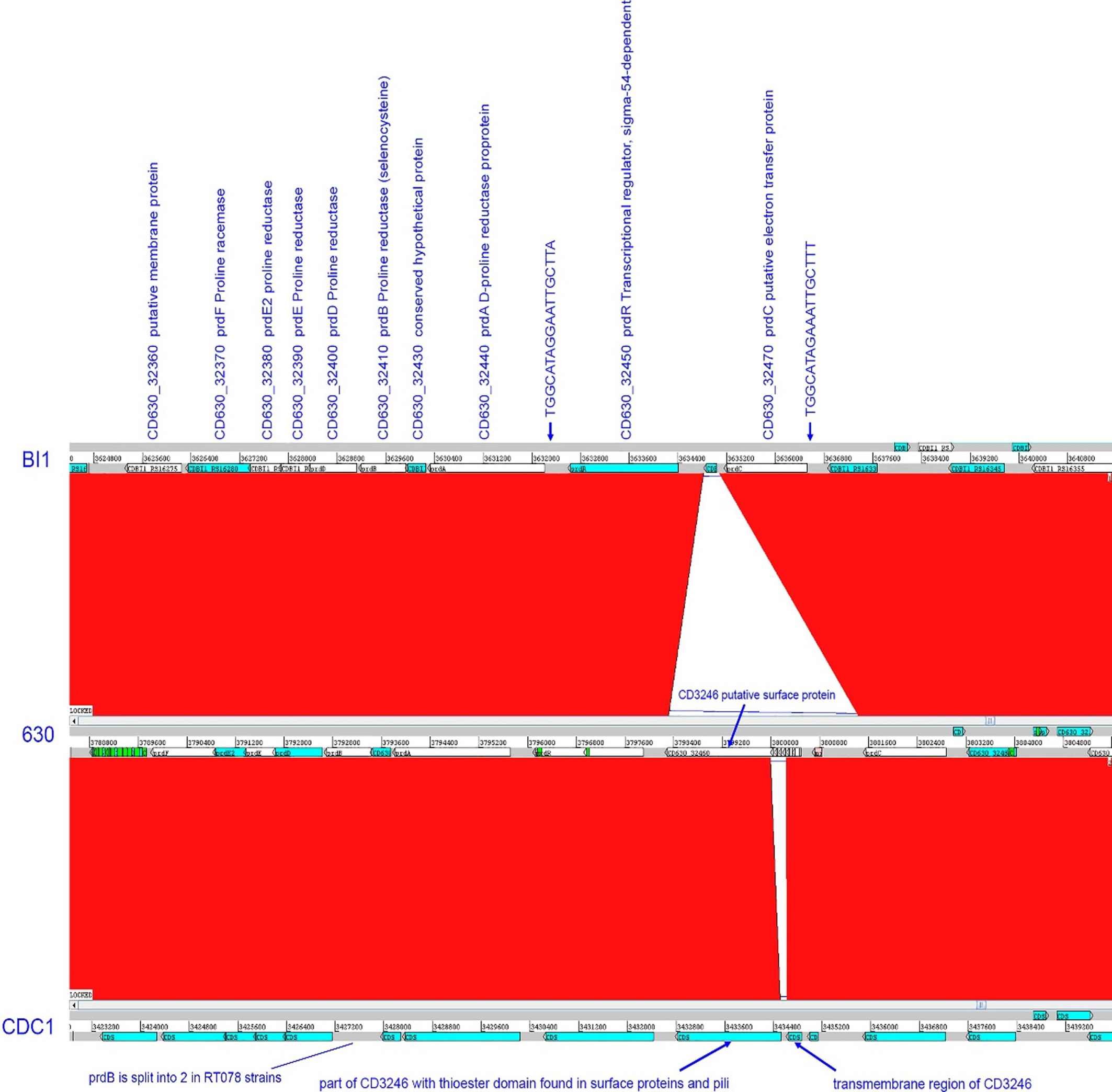

Supplement: Supplementary file 3 [file Image_3.TIF]

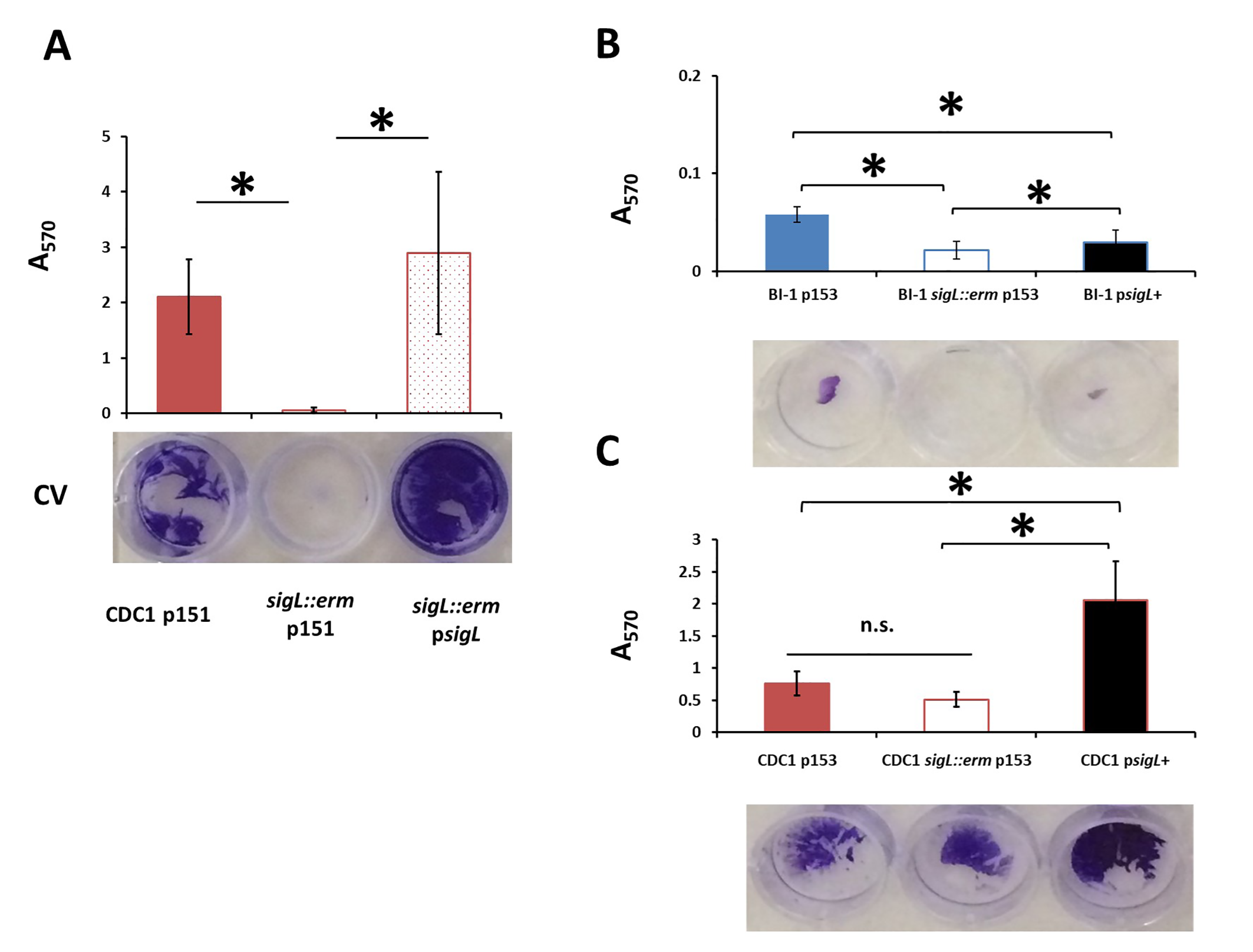

Supplement: Supplementary file 4 [file Image_4.TIF]

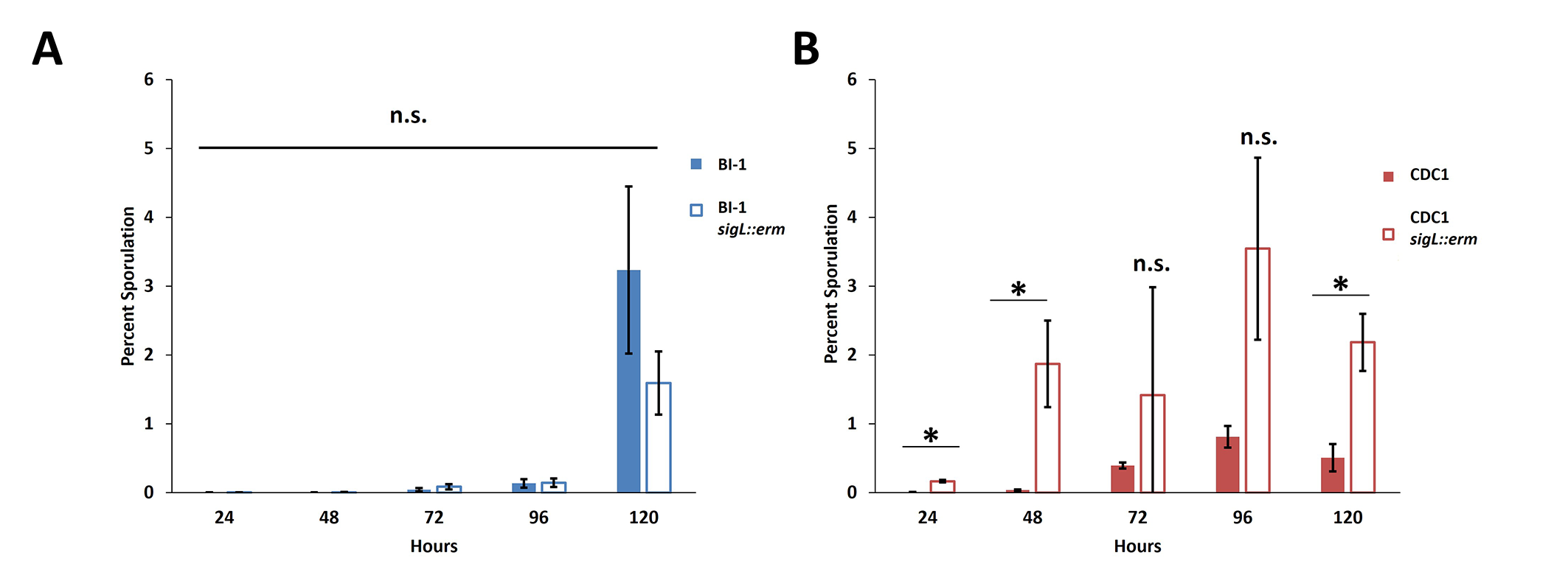

Supplement: Supplementary file 5 [file Image_5.TIF]

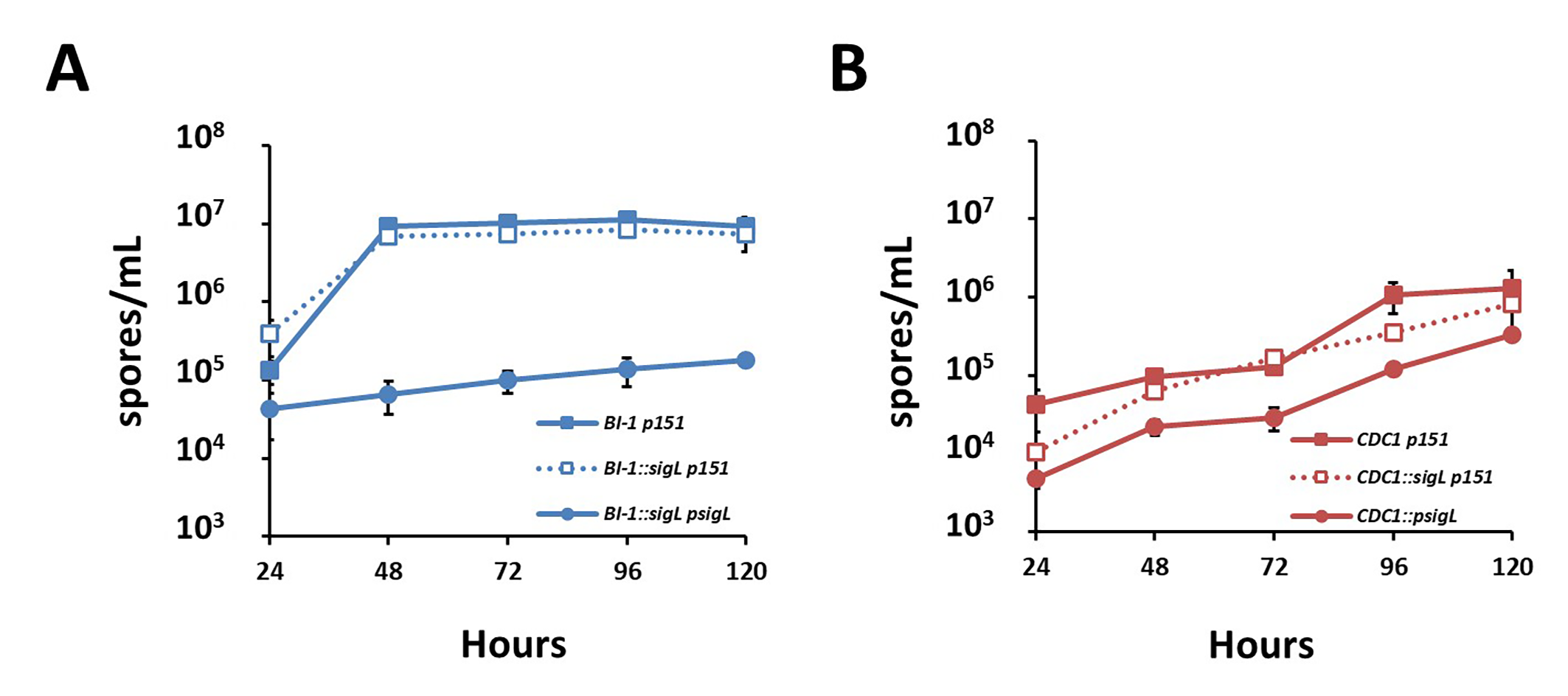

Supplement: Supplementary file 6 [file Image_6.TIF]

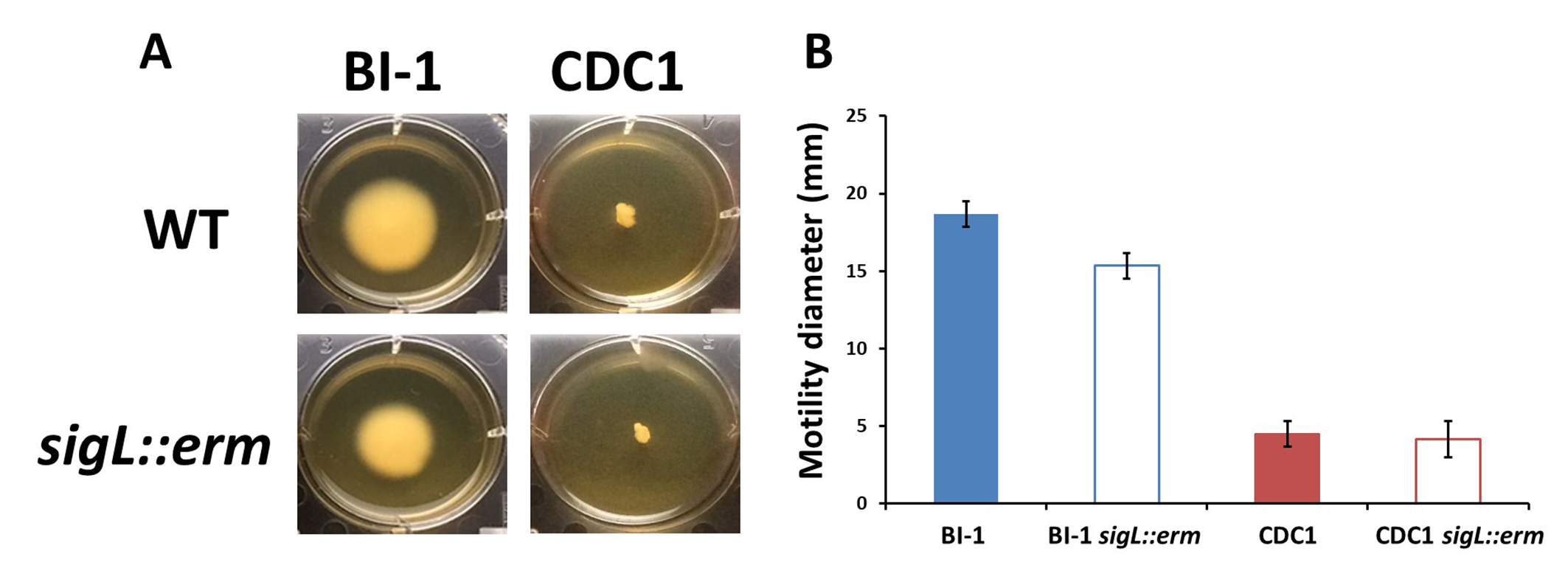

Supplement: Supplementary file 7 [file Image_7.TIF]

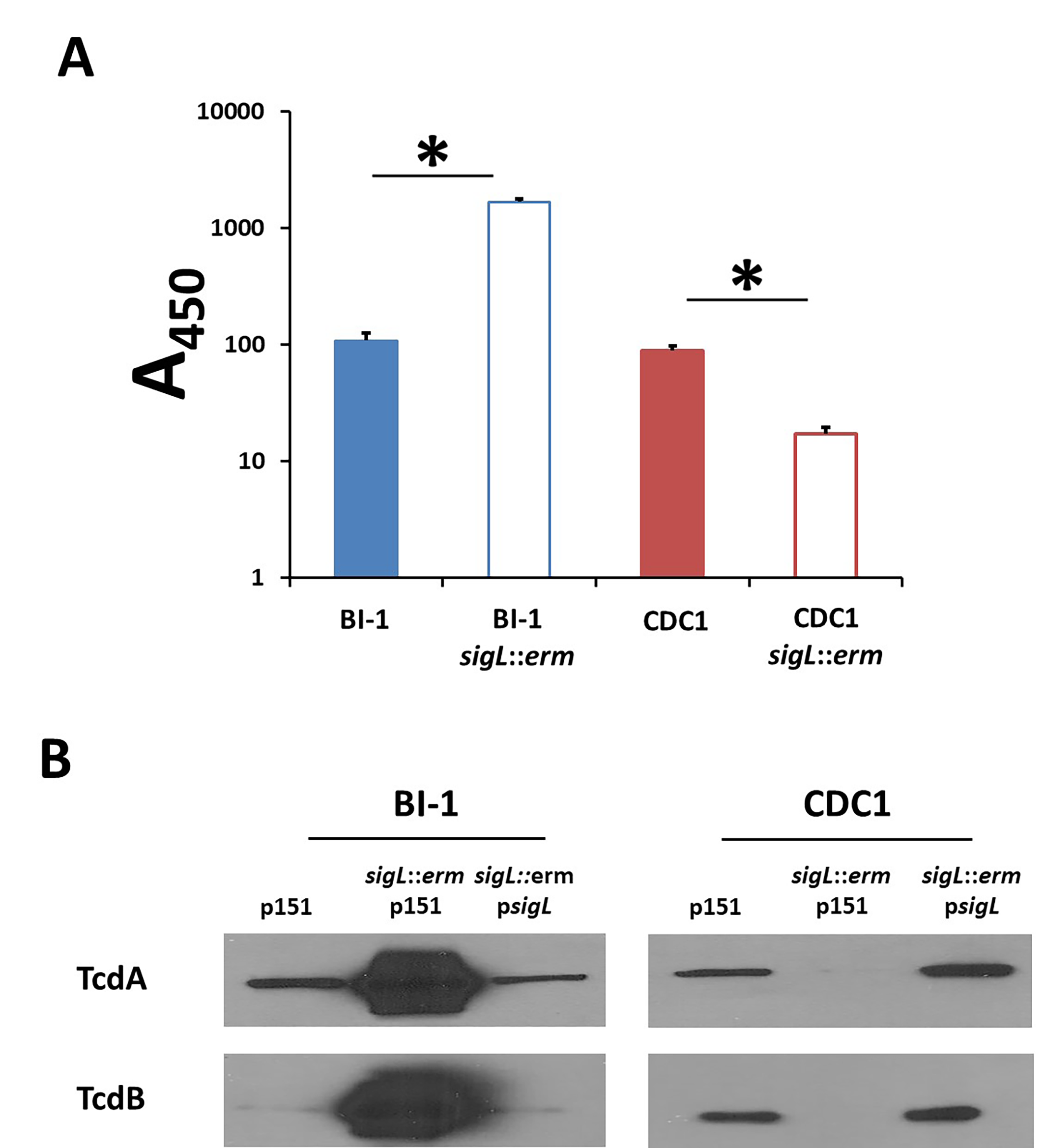

Supplement: Supplementary file 8 [file Image_8.TIF]
