# Supplementary material for: The Alternative Sigma Factor SigL Influences Clostridioides difficile Toxin Production, Sporulation, and Cell Surface Properties
Source: Front Microbiol. 2022 May 11;13:871152. doi: 10.3389/fmicb.2022.871152 (PMC9130780; doi:10.3389/fmicb.2022.871152)
Supplement: Supplementary file 12 [file Presentation_1.PPTX]

## Slide 1
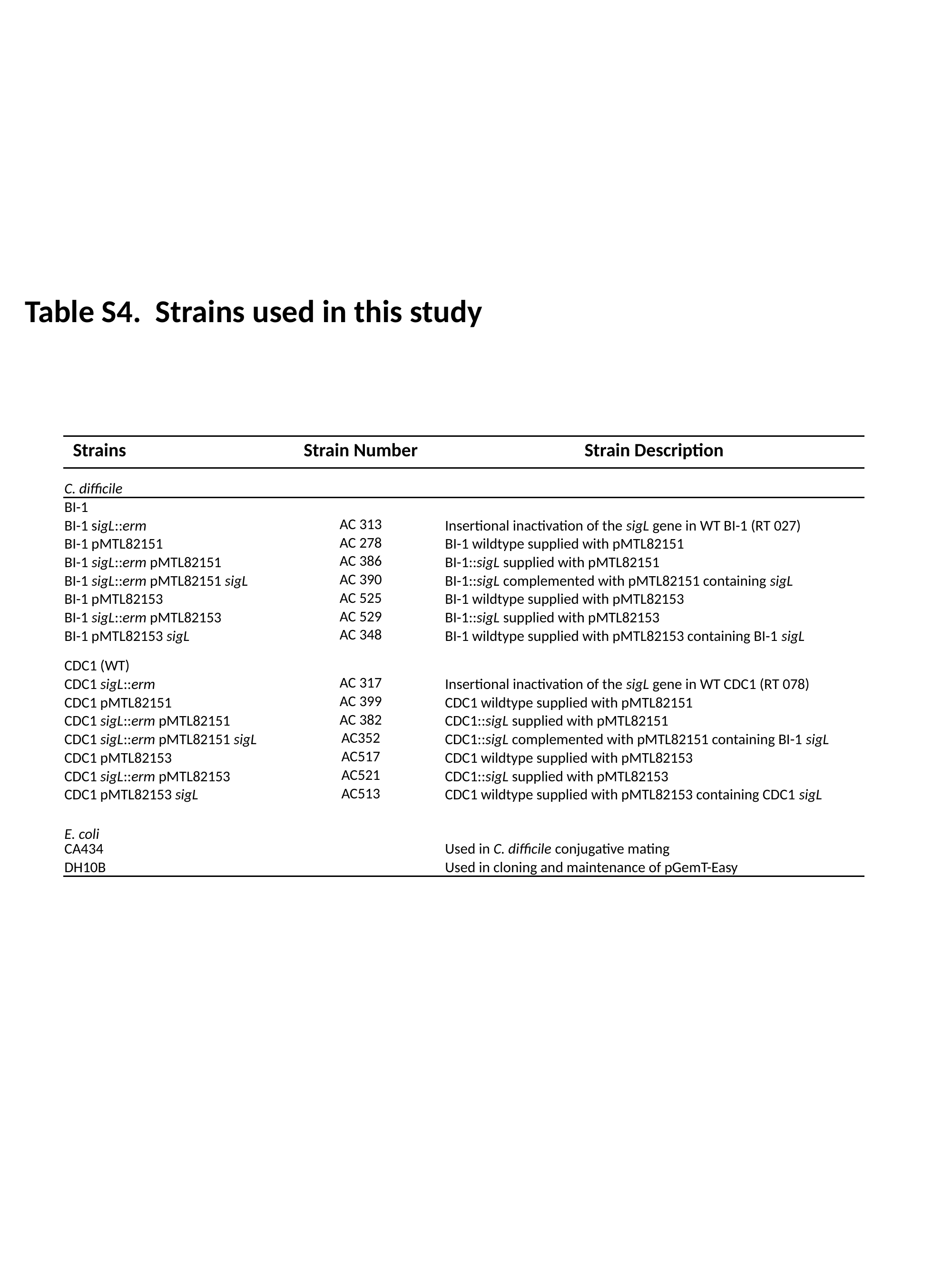

Table S4. Strains used in this study
| Strains | Strain Number | Strain Description |
| --- | --- | --- |
| C. difficile | | |
| BI-1 | | |
| BI-1 sigL::erm | AC 313 | Insertional inactivation of the sigL gene in WT BI-1 (RT 027) |
| BI-1 pMTL82151 | AC 278 | BI-1 wildtype supplied with pMTL82151 |
| BI-1 sigL::erm pMTL82151 | AC 386 | BI-1::sigL supplied with pMTL82151 |
| BI-1 sigL::erm pMTL82151 sigL | AC 390 | BI-1::sigL complemented with pMTL82151 containing sigL |
| BI-1 pMTL82153 | AC 525 | BI-1 wildtype supplied with pMTL82153 |
| BI-1 sigL::erm pMTL82153 | AC 529 | BI-1::sigL supplied with pMTL82153 |
| BI-1 pMTL82153 sigL | AC 348 | BI-1 wildtype supplied with pMTL82153 containing BI-1 sigL |
| CDC1 (WT) | | |
| CDC1 sigL::erm | AC 317 | Insertional inactivation of the sigL gene in WT CDC1 (RT 078) |
| CDC1 pMTL82151 | AC 399 | CDC1 wildtype supplied with pMTL82151 |
| CDC1 sigL::erm pMTL82151 | AC 382 | CDC1::sigL supplied with pMTL82151 |
| CDC1 sigL::erm pMTL82151 sigL | AC352 | CDC1::sigL complemented with pMTL82151 containing BI-1 sigL |
| CDC1 pMTL82153 | AC517 | CDC1 wildtype supplied with pMTL82153 |
| CDC1 sigL::erm pMTL82153 | AC521 | CDC1::sigL supplied with pMTL82153 |
| CDC1 pMTL82153 sigL | AC513 | CDC1 wildtype supplied with pMTL82153 containing CDC1 sigL |
| | | |
| E. coli CA434 | | Used in C. difficile conjugative mating |
| DH10B | | Used in cloning and maintenance of pGemT-Easy |
