# Supplementary material for: The Alternative Sigma Factor SigL Influences Clostridioides difficile Toxin Production, Sporulation, and Cell Surface Properties
Source: Front Microbiol. 2022 May 11;13:871152. doi: 10.3389/fmicb.2022.871152 (PMC9130780; doi:10.3389/fmicb.2022.871152)
Supplement: Supplementary file 13 [file Presentation_2.PPTX]

## Slide 1
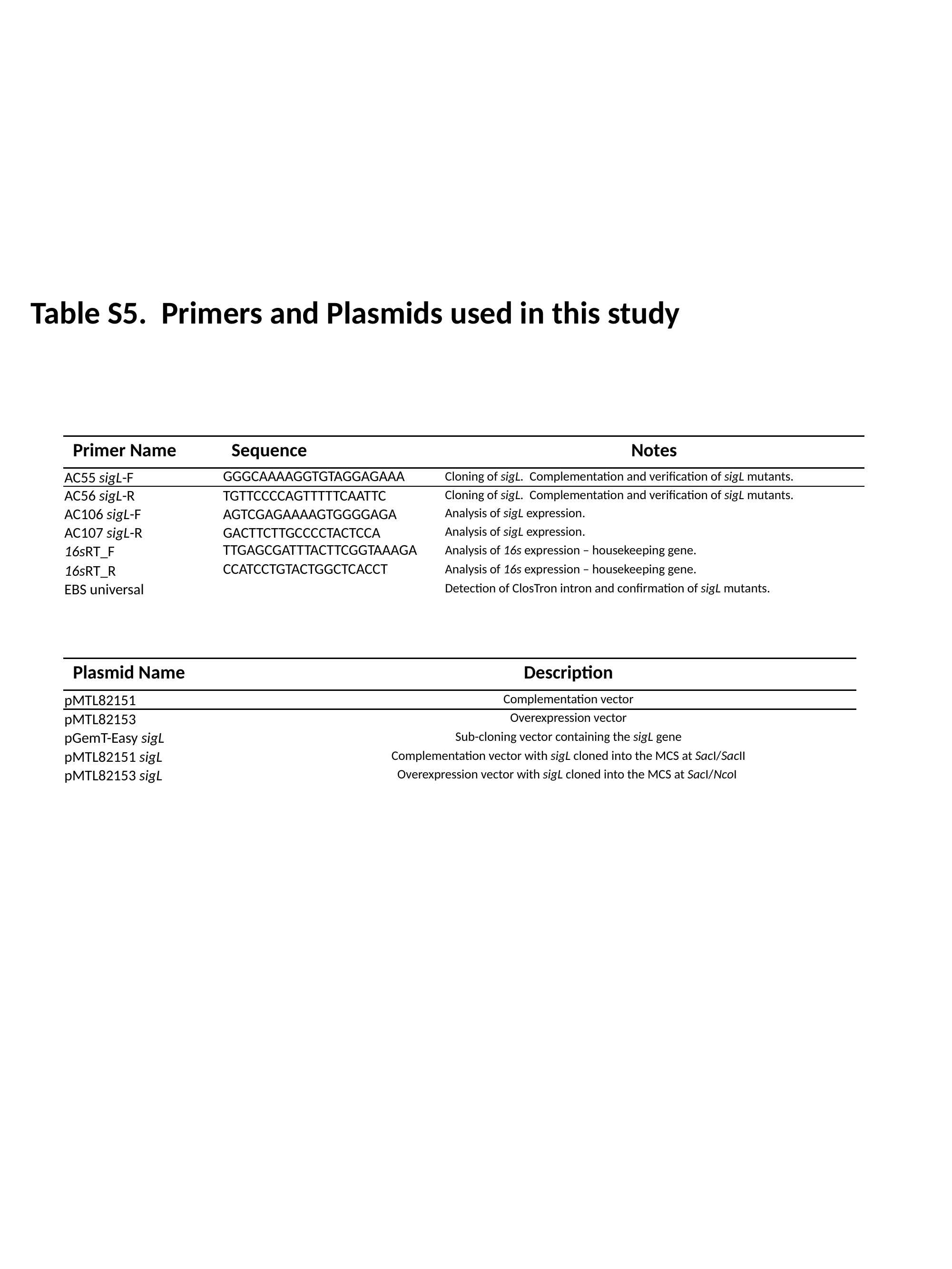

Table S5. Primers and Plasmids used in this study
| Primer Name | Sequence | Notes |
| --- | --- | --- |
| AC55 sigL-F | GGGCAAAAGGTGTAGGAGAAA | Cloning of sigL. Complementation and verification of sigL mutants. |
| AC56 sigL-R | tgttccccagtttttcaattc | Cloning of sigL. Complementation and verification of sigL mutants. |
| AC106 sigL-F | agtcgagaaaagtggggaga | Analysis of sigL expression. |
| AC107 sigL-R | gacttcttgcccctactcca | Analysis of sigL expression. |
| 16sRT\_F | TTGAGCGATTTACTTCGGTAAAGA | Analysis of 16s expression – housekeeping gene. |
| 16sRT\_R | CCATCCTGTACTGGCTCACCT | Analysis of 16s expression – housekeeping gene. |
| EBS universal | | Detection of ClosTron intron and confirmation of sigL mutants. |
| | | |
| Plasmid Name | Description |
| --- | --- |
| pMTL82151 | Complementation vector |
| pMTL82153 | Overexpression vector |
| pGemT-Easy sigL | Sub-cloning vector containing the sigL gene |
| pMTL82151 sigL | Complementation vector with sigL cloned into the MCS at SacI/SacII |
| pMTL82153 sigL | Overexpression vector with sigL cloned into the MCS at SacI/NcoI |
